# The new insight into the inflammatory response following focused ultrasound-mediated blood–brain barrier disruption

**DOI:** 10.1186/s12987-022-00402-3

**Published:** 2022-12-23

**Authors:** Hyo Jin Choi, Mun Han, Hyeon Seo, Chan Yuk Park, Eun-Hee Lee, Juyoung Park

**Affiliations:** 1 Medical Device Development Center, Daegu-Gyeongbuk Medical Innovation Foundation (K-MEDI Hubub), 80, Cheombok-Ro, Dong-Gu, Daegu, 41061 Republic of Korea; 2 Department of Computer Science, Gyeongsang National University, 501, Jinju-Daero, Jinju, Gyeongsangnam-Do 52828 Republic of Korea; 3 College of Future Industry, Department of High-Tech Medical Device, Gachon University, 1342, Seongnam-Daero, Sujeong-Gu, Seongnam, Gyeonggi 13120 Republic of Korea

**Keywords:** Blood–brain barrier, Focused ultrasound, Acute neuroinflammation, Genome profiling, Astrocyte

## Abstract

**Background:**

Despite the great potential of FUS-BBB disruption (FUS-BBBD), it is still controversial whether FUS-BBBD acts as an inducing factor of neuro-inflammation or not, and the biological responses after FUS-BBBD triggers the inflammatory process are poorly understood. The aim of this study is to investigate the safety window for FUS levels based on a comprehensive safety assessment.

**Methods:**

The mice were treated with two different ultrasound parameters (0.25 MPa and 0.42 MPa) in the thalamus region of brain. The efficacy of BBB opening was verified by dynamic contrast-enhanced MRI (DCE-MRI) and the cavitation monitoring. The transcriptome analysis was performed to investigate the molecular response for the two BBBD conditions after FUS-mediated BBB opening in time-dependent manners. Histological analysis was used for evaluation of the tissue damage, neuronal degeneration, and activation of glial cells induced by FUS-BBBD.

**Results:**

The BBBD, as quantified by the *K*_*trans*_, was approximately threefold higher in 0.42 MPa-treated group than 0.25 MPa-treated group. While the minimal tissue/cellular damage was found in 0.25 MPa-treated group, visible damages containing microhemorrhages and degenerating neurons were detected in 0.42 MPa-treated group in accordance with the extent of BBBD. In transcriptome analysis, 0.42 MPa-treated group exhibited highly dynamic changes in the expression levels of an inflammatory response or NF-κB pathway-relative genes in a time-dependent manner whereas, 0.25 MPa was not altered. Interestingly, although it is clear that 0.42 MPa induces neuroinflammation through glial activation, neuroprotective properties were evident by the expression of A2-type astrocytes.

**Conclusions:**

Our findings propose that a well-defined BBBD parameter of 0.25 MPa could ensure the safety without cellular/tissue damage or sterile inflammatory response in the brain. Furthermore, the fact that the excessive sonication parameters at 0.42 MPa could induce a sterile inflammation response via glial activation suggested the possibility that could lead to tissue repair toward the homeostasis of the brain microenvironment through A2-type reactive astrocytes.

**Supplementary Information:**

The online version contains supplementary material available at 10.1186/s12987-022-00402-3.

## Introduction

Focused ultrasound (FUS) combined with microbubbles (MBs) is a promising medical tool that can help therapeutic agents penetrate the temporarily disrupted blood–brain barrier (BBB) in various brain disorders [1]. FUS can induce mechanical stress by oscillation of MBs within a highly specialized vasculature and disrupt the BBB in the desired brain region [2]. The FUS-BBB disruption (FUS-BBBD) technique has been validated and optimized in various experimental models, from rodents [3–5] to non-human primates (NHPs) [6, 7]. Currently, clinical trials are underway, including those for Alzheimer’s disease (AD) (NCT03739905, NCT03671889, NCT04118764) [8], glioblastoma (NCT04063514) [9], and Parkinson’s disease (PD) (NCT04692116) [10]. More recently, based on data from pivotal clinical trials, the US Food and Drug Administration approved the clinical FUS device in 2021 to include patients with advanced PD that have mobility, rigidity, or dyskinesia symptoms [10].

Despite the powerful aspects of FUS-BBBD, its potentially detrimental effects are expected to have safety issues. Several studies have shown that FUS-BBBD induces intracerebral hemorrhage, transient edema, cell death, and glial activation according to the intensity of the exposure [11–13]. Safety should be considered when inducing vascular permeability using the FUS-BBBD system to minimize the risk of brain damage. The FUS parameters are the primary factors that alter the biological response to FUS-BBBD. Acoustic frequencies ranging from 28 kHz to 8 MHz have been used to increase the permeability of the BBB [14, 15]. Previous studies have shown that ultrasound intensity can affect the extent of BBBD, and the classification of tissue hemorrhages was used to grade ultrasound pressure [16]. Some reports have shown that factors such as pulse length, pulse repetition frequency (PRF), and sonication duration also influence the biological outcomes of sonication [17–19]. It is essential to consider that the sonication parameters should be well-designed to balance the enhancements of BBB permeability and have an acceptable impact on tissue damage.

Next, MBs play an essential role in the safety profile of FUS-BBBD. Even if used for equal FUS-BBBD protocols, MB responses could induce an unexpected reaction, depending on the dose, size distribution, and shell composition of MB [20–22]. Acoustic emissions from MB oscillations during sonication can be used to assess the activity of MBs in vivo. At sufficient pressures, stable cavitation generated by stably oscillating MBs could induce the BBB permeability with minimal negative effects on the target sites. Meanwhile, inertial cavitation could be generated by the collapse of MBs in the further increase in the ultrasound waves. Recently, acoustic cavitation monitoring systems have been considered as new reliable safety indicators of BBB opening [23]. Several studies have reported a correlation between acoustic emission acquired with a passive cavitation detector (PCD) and the outcome of FUS-BBBD treatment [12, 24]. This control system maintains the acoustic pressure within the threshold and enables effective BBBD and drug delivery without causing visible tissue damage in rodents [25, 26] and NHPs [27].

The main safety concern for FUS-BBBD is the inflammatory response in the BBB-opening region. A recent study suggested that FUS-BBBD alters the parenchymal microenvironment by increasing pro-inflammatory cytokines, damage-associated molecular patterns (DAMPs), and cell adhesion molecules [28]. These acute inflammatory molecules induce sterile inflammation, eliciting tissue damage and repair [16, 29]. In particular, the potential of FUS-BBBD has been demonstrated in the clearance of amyloid plaques in AD models based on the innate/adaptive immune response [30, 31]. Recent transcriptomic studies have shown that FUS-mediated inflammation depends on microbubble dose, cavitation, and FUS parameters [26, 32, 33]. Furthermore, Mathew et al. suggested that the diversity of anesthetics affects the underlying reactivity in brain tissues after FUS-BBBD [34]. Whether inflammation-mediated FUS-BBBD is harmful remains controversial, and the knowledge of the mechanisms underlying and beyond the inflammation-mediated biological response to the contributions of FUS experimental parameters is limited.

Microglia and astrocytes serve as sensors of events within the central nervous system (CNS) and contribute to neuroinflammation progression [35]. Microglia differentiate into two different phenotypes after activation, termed “M1” and “M2,” based on neuroinflammation and ischemia [36, 37]. This terminology also parallels the “A1” and “A2” reactive astrocyte [38, 39]. The functional phenotype of M1- and A1-like glial cells is characterized by the upregulation of inflammatory response molecules, which could have a harmful function. In contrast, M2 and A2 glial cells upregulate the expression of immunosuppressive and neuroprotective molecules, suggesting their beneficial and restorative functions. Consistent with this, A2-reactive astrocytes have been reported to exert beneficial effects by upregulating many neurotrophic factors, leading to recovery and repair of the CNS after damage [40–43]. Although recent studies have reported the activation of neuroglial cells in response to FUS-BBBD [13, 28, 44], the relationship between glial cell polarization and ultrasound-mediated mechanical bioeffects remain unclear. In this study, we suggest that FUS-BBBD induces alterations in the glial cell subtype.

The safety of FUS-BBBD is sensitive to a multitude of various factors. To better understand the safety window after FUS application, it is necessary to simultaneously assess both commonly assessed methodologies (e.g. MR imaging, histology, and cavitation monitoring) and molecular biological reactivity (e.g., transcriptome screening). Therefore, this study aimed to provide a comprehensive safety profile, evaluated by transcriptome analysis, along with different BBB permeability and cavitation activity after two different acoustic pressures. Furthermore, to explore the effects of the homeostatic response after FUS-BBBD, transcriptome profiling was performed based on their subtypes of activated glial cells. This study provides new insights into the inflammatory response in the brain following FUS-BBBD.

## Materials and methods

### Animals

All experiments were conducted following the procedure approved by the Institutional Animal Care and Use Committee (IACUC) at the Daegu-Gyeongbuk Medical Innovation Foundation (DGMIF-19100701-00). Eight-week-old male Institute of Cancer Research (ICR) mice (Orient Bio Inc., Seongnam, Korea) were anesthetized using a mixture of Zoletil 25 mg/kg (Virbac, Carros, France) and Rompun (4.6 mg/kg; Bayer, Leverkusen, Germany) that were administered intramuscularly and were constantly monitored throughout the experiment. A total of 90 ICR mice were randomly divided into five groups for experimental purposes: characterization of BBB permeability and passive cavitation detection (n = 12), transcriptome and qRT-PCR analysis (n = 24), western blot analysis (n = 24), immunofluorescence analysis (n = 24), and hematoxylin and eosin (H&E) histopathology (n = 6).

### Characterization of blood–brain barrier permeability

The pre-clinical MRgFUS system (RK-100, FUS Instrument, Toronto, Canada) was used for BBB disruption, as described previously [45]. Briefly, the device comprises an air-backed, single-element, spherically curved piezoelectric transducer (FUS Instrument, Toronto, Canada) with a diameter of 75 mm and radius of curvature of 60 mm, and a resonant frequency of 1.1 MHz. The distribution of ultrasound pressure at the free water’s focal region was measured using an acoustic intensity measurement system (AIMS III, ONDA Sunnyvale, CA, USA) with a hydrophone (HGL-400, ONDA). The transducer was submerged in a water tank filled with degassed water. The animal was placed on an MR-compatible animal bed in a supine position, as shown in Additional file 1: Figure S1. Then, 9.4 T pre-clinical MRI (BioSpec 94/20 USR, Bruker, Ettlingen, Germany) was used for image guidance for the focused ultrasound system and BBB permeability characterization. A radiofrequency coil with an inner diameter of 86 mm was used for the signal transmission. 2D rapid acquisition with refocused echoes (RARE) pulse sequence was used for the acquisition of T2-weighted images, which were used as an image guide with the parameters set as described in Additional file 1: Table S8. MRgFUS was applied to four targets in the whole thalamus region for transcriptome/immunoblot analysis (Additional file 1: Figure S2a) or two targets in the thalamus region of one hemisphere for immunohistochemistry analysis (Additional file 1: Figure S2b). Based The son the MR image, the only FUS-targeted thalamus region from the entire brain was isolated, and molecular-based experiments were further conducted. Before the FUS application, activated microbubbles (0.02 mL/kg, Definity, Lantheus Medical Imaging, North Billerica, MA, USA) were diluted 1:50 in normal saline injected through a tail vein catheter using an automated syringe pump (Harvard Apparatus, Holliston, MA, USA). Subsequently, to disrupt the BBB, a 10 ms burst sonication at 0.25-or 0.42 MPa acoustic peak pressure measured in a free water condition with a 1 Hz pulse repetition frequency (PRF) for 120 s (duty cycle: 1%) was delivered to the thalamus area of the rat brain. After BBB disruption, a rapid acquisition with refocused echoes variable repetition time (RARE VTR) and dynamic contrast-enhanced (DCE)-MRI [45] or T1-weighted MR images with the parameters set as described in Additional file 1: Table S8 were acquired with a 0.02 ml/kg gadolinium-based contrast agent (Dotarem, Guerbet, Roissy, France) injected through a tail vein to confirm BBB permeabilization. The BBB permeability (*K*_*trans*_) was estimated using the Patlak model [46] using DCE-MRI images. For further experiments, the brain was transcardially perfused with 0.9% NaCl at the indicated time points, followed by 4% paraformaldehyde (PFA) fixation or snap-freezing.

### Passive cavitation detection

The acoustic cavitation was acquired from a passive cavitation detector (PCD; V306, center frequency: 2.25 MHz, OLYMPUS, MA, USA) inserted in the transducer center for sonication. The acquired signals were transmitted to the DAQ board (ATS460, AlazarTech, Quebec, Canada) in the MRgFUS system, and sampling was conducted at 20 MHz, 14 bit, and 125 MS/s. The emission signals recorded during BBBD were normalized to the base signal without MB at the same locations. The cavitation dose was calculated based on the integrated area under the curve of the temporal power variance of the emission signals monitored during sonication. Three cavitation parameters that characterize the cavitation behaviors were calculated: stable cavitation dose with harmonic (SCD_h_), stable cavitation dose with subharmonics and ultraharmonics (SCD_u_), and inertial cavitation dose with broadband emission (ICD) [47, 48]. The stable harmonic components were identified as the peak value around each harmonic (n*f*_*c*_, n = 1, 2, 3…*f*_c_ = 1.1 MHz) and sub-/ultra-harmonic (n*f*_c_/2, n = 1, 3, 5…) frequencies.

### cDNA library construction and RNA sequencing

For the assessment of gene expression levels, the mice were sacrificed at 1, 6, 12, 24, and 48 h after BBBD (n = 2 per time point). The sham control mice received MB and contras agents but without FUS sonication (n = 2). The tissue was snap-frozen in liquid nitrogen and stored at− 80 °C until RNA isolation. According to the manufacturer’s instructions, tissues (100–120 mg) were homogenized and isolated using the RNeasy Mini Kit (Qiagen, Hilden, Germany). The total RNA concentration and quality were determined using a NanoDrop™ 2000 spectrophotometer (Thermo Fisher Scientific, Waltham, MA, USA). Subsequently, total RNA was used for sequencing or real-time qPCR analyses. cDNA libraries were prepared with 1 μg of total RNA using the Illumina TruSeq RNA Sample Prep Kit v2 (Illumina Inc., San Diego, CA, USA). Next, paired-end sequencing was performed using the Illumina HiSeq^™^4000 sequencing instrument, according to the manufacturer’s instructions, yielding 100-bp paired-end reads.

### Transcriptome assembly and analysis

Reads were mapped to the genomic DNA reference (UCSC mm10) using HISAT2 version 2.1.0 [49] and Bowtie2 2.3.4.1 [50]. Subsequently, String Tie version 1.3.4d [51] was used to perform transcript assembly with aligned reads. The transcript levels of each unigene were determined by the total mapped read numbers and normalized to detect fragments per kilobase of exon per million fragments mapped (FPKMs). Reads that included genes with FPKM values of 0 in all samples for each gene were excluded from the analysis. For differential expression genes (DEGs) analysis, the values of log2 (FPKM + 1) were calculated for quantile normalization. Gene ontology (GO) enrichment analysis was performed using the g:Profiler tool (https://biit.cs.ut.ee/gprofiler/) for the gene list with significant expression level differences. For functional annotation, analysis of pathway enrichment based on the KEGG pathway (http://www.kegg.jp/kegg/pathway.html) was performed and visualized as a heatmap. Raw data were assessed for statistical significance with a threshold of *P* < 0.05, between fold change by using an independent *t*-test.

### Real-time quantitative reverse transcription PCR analysis

PCR primers were designed based on the transcriptome sequence using the Primer 3 software (ver. 4.0; http://primer3.ut.ee). cDNA was synthesized from 2 μg of total RNA using the Omniscript RT kit (Qiagen), according to the manufacturer’s instructions. GAPDH served as an internal control. Real-time qRT-PCR was performed using a Power SYBR™ Green PCR Master Mix (Thermo Fisher Scientific) on an Applied Biosystems 7500 Real-Time PCR Instrument System (Thermo Fisher Scientific). The primer sequences are listed in Additional file 1: Table S7. The real-time PCR program was 95 °C for 2 min, followed by 40–45 cycles of 95 °C for 15 s, 60 °C for 15 s, and 72 °C for 20 s. Melting curve analysis was performed at the end of cycling to ensure single product amplification of the appropriate melting temperature using the Applied Biosystems ABI 7500 Software (ver. 2.3; Thermo Fisher Scientific) (Additional file 1: Figure S6). The difference in the cycle threshold (Ct) value of the difference between the target gene and its housekeeping gene (*GAPDH*) was calculated using the 2^−ΔΔCt^ method [52]. Experiment description and data presentation follow the guidelines on the minimal information for publication of quantitative PCR experiments [53].

### Western blot analysis

Western blot analysis was performed on proteins isolated from the brain tissues at 1, 6, 12, 24, and 48 h after BBBD (n = 2, per time point). The brain tissues were lysed in RIPA lysis buffer (Thermo Fisher Scientific) containing a protease/phosphatase inhibitor cocktail (Cell Signaling Technology, Danvers, MA, USA). Total protein concentration was determined using the Pierce™ BCA Protein Assay Kit (Thermo Fisher Scientific). Equal amounts of total protein were loaded onto 12% sodium dodecyl sulfate–polyacrylamide gel electrophoresis and transferred to a pre-activated polyvinylidene fluoride (PVDF) membrane (Bio-Rad, Hercules, CA, USA). The PVDF membrane was blocked with 5% non-fat skim milk in Tris-buffered saline (TBS; pH 7.4) containing 0.1% Tween 20 for 1 h at room temperature. Blots were incubated overnight at 4 °C with rabbit anti-p65 (Cell Signaling, #4764; 1:1000), rabbit anti-phospho-p65 (Cell Signaling, #3033; 1:500), rabbit anti- IκBα (Cell Signaling, #4812; 1: 1000), mouse anti-phospho-IκBα (Cell Signaling, #9246; 1:500), and rabbit anti-GAPDH (Cell Signaling, #2118; 1: 5000). Subsequently, the blots were incubated with HRP-conjugated secondary antibodies at 1: 3000 for 1 h at room temperature and developed using ECL Prime western blotting Detection Reagent (GE Healthcare, Buckinghamshire, UK). Visualization and imaging of the blots were performed using a ChemiDoc MP imaging system (Bio-Rad).

### Immunofluorescence analysis

The mice were sacrificed at 1 h, 6 h, 12 h, 24 h, and 48 h after BBBD (n = 2 per time point). To perform the immunofluorescence assay, mice were transcardially perfused with 0.9% NaCl and ice-cold 4% formaldehyde. Extracted brains were post-fixed with 4% formaldehyde for 3 days at 4 °C. Fixed brains were sliced into 30 μm sections using a vibrating blade microtome (Leica VT1200S, Leica Microsystems, Wetzlar, Germany). Tissue slices were permeabilized with 0.2% Triton X-100 (Sigma-Aldrich, St. Louis, MO, USA) for 1 h at room temperature. The tissues were incubated for 2 h in a blocking solution containing 10% normal goat serum (Abcam, Cambridge, MA, USA) followed by overnight incubation with specific primary antibodies, including rabbit anti-Iba‐1 (Wako, Osaka, Japan, #019-19741; 1:250) and mouse anti-GFAP (Sigma-Aldrich, #G3893; 1:1000). Secondary antibodies included Alexa Fluor 555 goat anti-rabbit IgG (Cell Signaling, #4413; 1: 1000), and Alexa Fluor 488 goat anti-mouse IgG (Cell Signaling, #4408; 1: 1000). The slides were mounted with a fluorescence mounting medium (Dako, Glostrup, Denmark).

### Histopathological staining

To assess tissue damage, mice were sacrificed approximately 4 h after sonication, and brain tissue was stained with hematoxylin and eosin (H&E) (n = 3) [29]. All tissue samples were fixed in 10% formalin for 3 days after resection with 3 mm thickness and embedded in paraffin using standard procedures. Serial sections of 6 μm thickness were prepared from each tissue and stained with a hematoxylin and eosin stain kit (VECTOR Laboratories, Burlingame, CA, USA). To observe neurons degenerated by sonication, FJC staining was performed at 24 h post sonication (n = 2) using a commercial kit (Biosensis, Thebarton, South Australia). Briefly, tissue slides were immersed in 10% sodium hydroxide solution (v/v) for permeabilization, followed by incubation in 10% potassium permanganate solution (v/v) for blocking. A mixture of 20% FJC and 20% DAPI (v/v) was dropped onto the tissue slides and incubated for 20 min at room temperature. The slides were dried and covered with a coverslip using DPX mounting media (Sigma-Aldrich).

### Image analysis

The tissue slides from immunofluorescence and histological staining were imaged at 20 ×magnification using an Axio Scan.Z1 microscope (Carl Zeiss, Goettingen, Germany). The acquired images were processed using the Zen 2 image-processing software (blue edition, Carl Zeiss). To quantify immunofluorescence intensity of FJC staining, twenty ROIs in the sonicated region, which overlapping with axial plane of contrast-enhanced T1w MR images were selected in high magnification (×100) and analyzed using ImageJ software (version 1.40; National Institutes of Health, Bethesda, MD, USA).

### Statistical analysis

Statistical analysis was conducted using SPSS software (version 22.0, IBM Corp., NY, USA) and GraphPad Prism (version 8.0, GraphPad, CA, USA). All values are presented as mean ± SD. The means of the two groups were compared using a two-tailed Student’s unpaired *t*-test. Pearson’s correlation coefficient was used to evaluate the relationship between RNA sequencing and qPCR results. Statistical significance was set at *P* < 0.05.

## Results

### Confirmation of MRgFUS-induced BBBD

**Fig. 1:**
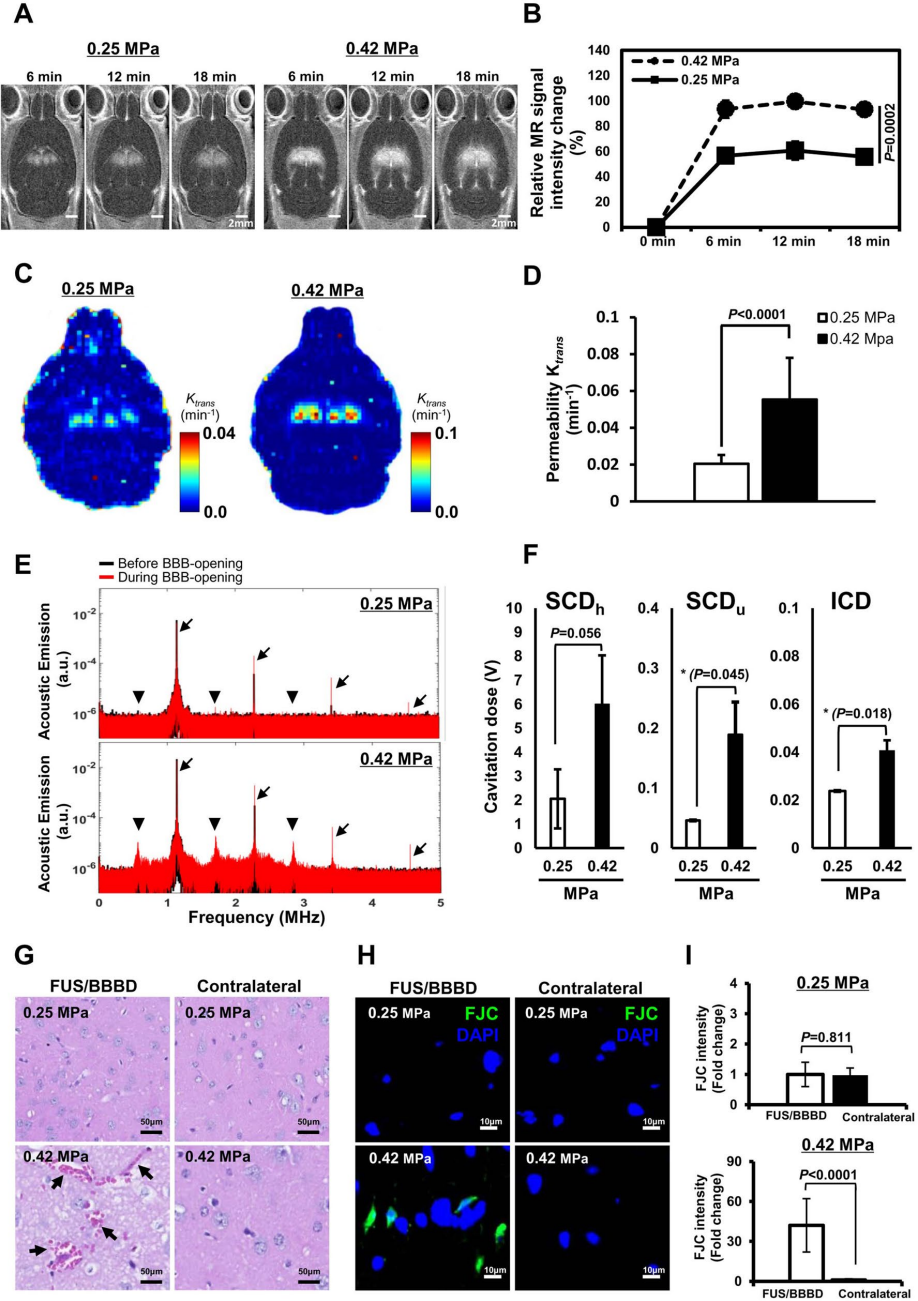
Establishment of BBBD conditions for the FUS parameters. (**A**) Representative contrast-enhanced T1 weighted MR images and relative MR signal intensity of contrast agents in the mouse brain after sonication with FUS parameters: 0.25 MPa (three left panels) and 0.42 MPa (three right panels). Time series (6, 12, and 18 min) of coronal MR images showed localized BBBD at four targeted points of the thalamus region and contrast changes. (**B**) The line graph illustrates the relative MR signal intensity corresponding to BBB permeability changes at the three time-points after contrast agent injection (n = 3, *P* = 0.0002; two-tailed Student’s *t*-test). Scale bars are 2 mm. (**C**–**D**) The *K*_*trans*_ mapping and mean values in the sonicated area of the hippocampus region for each FUS parameter are presented for comparison (n = 3, *P* < 0.0001; two-tailed Student’s *t*-test). (**E**) Representative acoustic emission spectra of before (black line) and after (red line) BBB-opening according to 0.25 MPa and 0.42 MPa. The black arrows indicate the locations of the fundamental frequency of the ultrasound and the second, third, and fourth harmonics. Arrowheads indicate subharmonics and ultraharmonics. (**F**) The bar graph presents the mean value of the cavitation dose calculated by integrating all spectra (n = 3, *P < 0.05; two-tailed Student’s *t*-test). (**G**–**H**) Histological analysis of the mouse brain after BBB opening with H&E (**G**) and FJC (**H**) staining. The black arrows in H&E stained tissue indicate petechiae (scale bar = 50 μm) (**G**). Degenerating neurons showed green fluorescence (FJC-positive cells, green color; DAPI, blue; scale bar = 10 μm) (**H**). (**I**) The intensity of FJC-positive cells measured and converted to relative intensity compared with the contralateral region (mean ± SD, multiple ROIs (n = 20) per hemisphere, *P* < 0.001; two-tailed Student’s *t*-test)

Two FUS experimental conditions were established using 0.25 and 0.42 MPa acoustic pressures to investigate the difference in the extent of BBB permeabilization by the ultrasound pressure. BBBD was confirmed by signal enhancement of MR contrast agent in T1-weighted MR images, after Gd-DTPA administration in a time-dependent manner (6, 12, and 18 min) (Fig. 1a). The relative MR signal intensity of the targeted BBBD region in the 0.25 MPa group increased to 56.6 ± 5.7%, while it increased to 93.2 ± 4.8% in the 0.42 MPa group (Fig. 1b).

We further investigated the permeability of the FUS-BBB opening using dynamic contrast-enhanced MRI (DCE-MRI) (Fig. 1c). In the 0.42 MPa group, the *K*_*trans*_ value was 0.06 ± 0.022 min^−1^, while in the 0.25 MPa group, *K*_*trans*_ reached a value of 0.02 ± 0.005 min^−1^. The *K*_*trans*_ in the 0.42 MPa group was approximately threefold higher than that in the 0.25 MPa ( *P*< 0.0001; two-tailed Student’s *t*-test, Fig. 1d). To compare the cavitation activity, cavitation doses were obtained from the acoustic emission spectra (Fig. 1e, f). The stable cavitation dose with harmonics (SCD_h_) signals were predominantly larger than those of stable cavitation dose with ultraharmonics (SCD_u_) or inertial cavitation dose (ICD) in both groups (Fig. 1e). As the acoustic pressure increased to 0.42 MPa, SCD_u_ increased, and an apparent ICD from broadband emission was observed compared to that in the 0.25 MPa group (Fig. 1f). These results suggest that the 0.42 MPa acoustic pressure condition induced a more permeable BBB than the 0.25 MPa condition via increased MB.

Next, we performed a histological assessment to observe the tissue/neuronal damage according to the extent of BBBD (Fig. 1g–i). Tissue subjected to 0.25 MPa FUS had no significant extravasated red blood cells (RBCs) (Fig. 1g; Additional file 1: Figure S3a) or FJC-positive neurodegeneration (Fig. 1h; Additional file 1: Figure S3c). In contrast, the tissue receiving FUS treatment at 0.42 MPa had regions of RBCs extravasation and microvacuolation (Fig. 1g; Additional file 1: Figure S3b), as well as FJC-positive neurodegeneration (Fig. 1h; Additional file 1: Figure S3d), with a 42-fold FJC intensity increase (*P* < 0.0001; two-tailed Student’s t-test, Fig. 1i). Our findings exhibited that the 0.25 MPa group showed sufficient BBBD without histological damage, while the 0.42 MPa group showed excessive BBBD with tissue/cellular damage.

### Transcriptional profiling after BBBD by two FUS-BBBD parameters

**Fig. 2:**
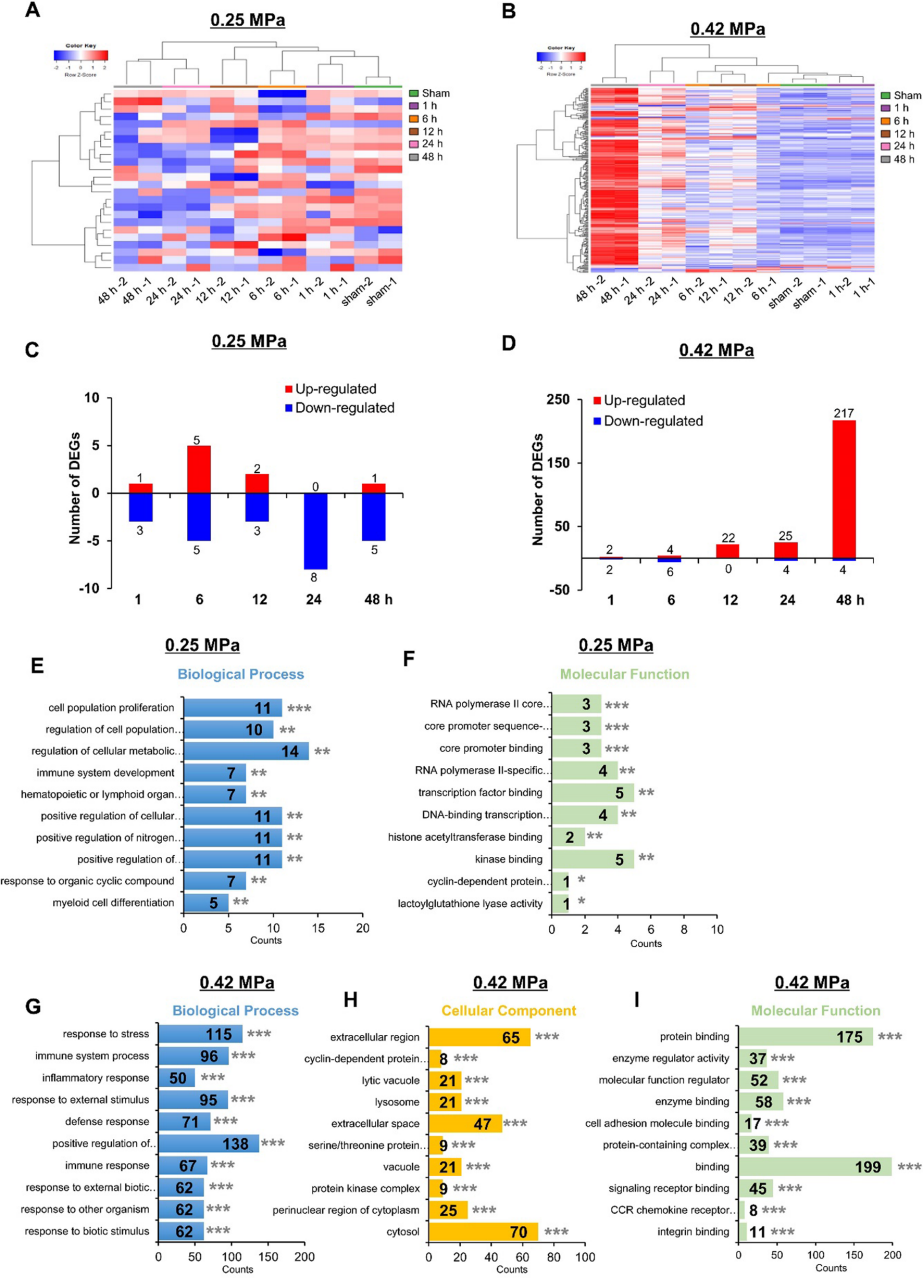
Transcriptome analysis of differentially expressed genes (DEGs) in the brain after FUS-BBBD. (**A**–**B**) Hierarchical clustering heat map with all the DEGs based on log2 FPKM values (fold-change > 2, *P* < 0.05; independent sample *t*-test, n = 2, per time point). The heat map indicates DEGs between the sham control and five samples of each time point after BBBD at 0.25 MPa (**A**) and 0.42 MPa (**B**). The red and blue colors indicate upregulation and downregulation, respectively (**C-D**) The number of DEGs between the sham control and each time point samples post-BBBD are shown as upregulated (red) or downregulated (blue) (fold-change > 2, *P* < 0.05). GO functional analysis of DEGs: 0.25 MPa (**E–F**) and 0.42 MPa **(G-I**). The distribution of GO terms of DEG was annotated in three ontology categories: biological process (blue; **E**, **G**), cellular component (yellow; **H**), and molecular function (green; **F**, **I**) (adjusted *p*-value; **P < 0.01, ***P < 0.001)

Given the differential tissue responses induced by two acoustic pressures, we performed transcriptome analysis to obtain a comprehensive profile of gene expression in the targeted BBB opening region by two FUS parameters in a time-dependent manner. Hierarchical clustering analyses of differentially expressed genes (DEGs) were performed following the criteria that satisfied both log2 (fold-change) > 2 and *P*-value < 0.05, in at least one of the total comparison pairs (sham control *versus* 1, 6, 12, 24, and 48 h) (Fig. 2a, b). In the 0.25 MPa group, 24 DEGs (0.14%) among the total 17,403 transcripts were identified in all comparisons (Fig. 2c; Additional file 1: Table S1), whereas 258 DEGs (1.47%) among the total 17,570 transcripts were identified in the 0.42 MPa group (Fig. 2d; Additional file 1: Table S2). Differences in the transcriptomes were observed, and the expression patterns of down-regulated DEGs were predominantly presented in the 0.25 MPa group following FUS-BBBD in a time-dependent manner (Fig. 2c). In contrast, the upregulated DEGs showed a remarkably increasing trend over time in the 0.42 MPa group (Fig. 2d). These results revealed that the 0.25 MPa condition had a weaker effect on gene expression than the 0.42 condition, implying that the 0.42 MPa condition could induce more complicated transcript regulation.

### Gene ontology (GO) analysis in two FUS-BBBD conditions

To investigate all molecular functions of DEGs, we performed GO functional classification for the 0.25 MPa and 0.42 MPa FUS parameters. We summarized the DEGs in the three GO domains: biological processes, cellular components, and molecular functions. All results were ranked by enrichment score, and the top 10 results of each category are shown in Fig. 2e–i. In the 24 DEGs of the 0.25 MPa group, “cell population proliferation” showed the most significantly enriched terms in the “biological process” domain (Fig. 2e). In terms of “molecular function,” genes associated with “RNA polymerase II core promoter sequence-specific DNA binding” most enriched (Fig. 2f). The “cellular component” domain showed no significant difference. At the 258 DEGs of 0.42 MPa condition, the stress response was highly enriched in the “biological process” category (Fig. 2g). In the “cellular component” domain, genes belonging to the “extracellular region” were highly enriched (Fig. 2h). “protein binding” was the top enriched term in the “molecular function” ontology (Fig. 2i). GO annotations were also analyzed by comparing five pairwise groups (sham control *vs.* 0.25 MPa and 0.42 MPa), and the 10 most enriched GO terms were obtained (Additional file 1: Figures S4, Additional file 1: S5).

### KEGG pathway enrichment analysis of DEGs

**Fig. 3:**
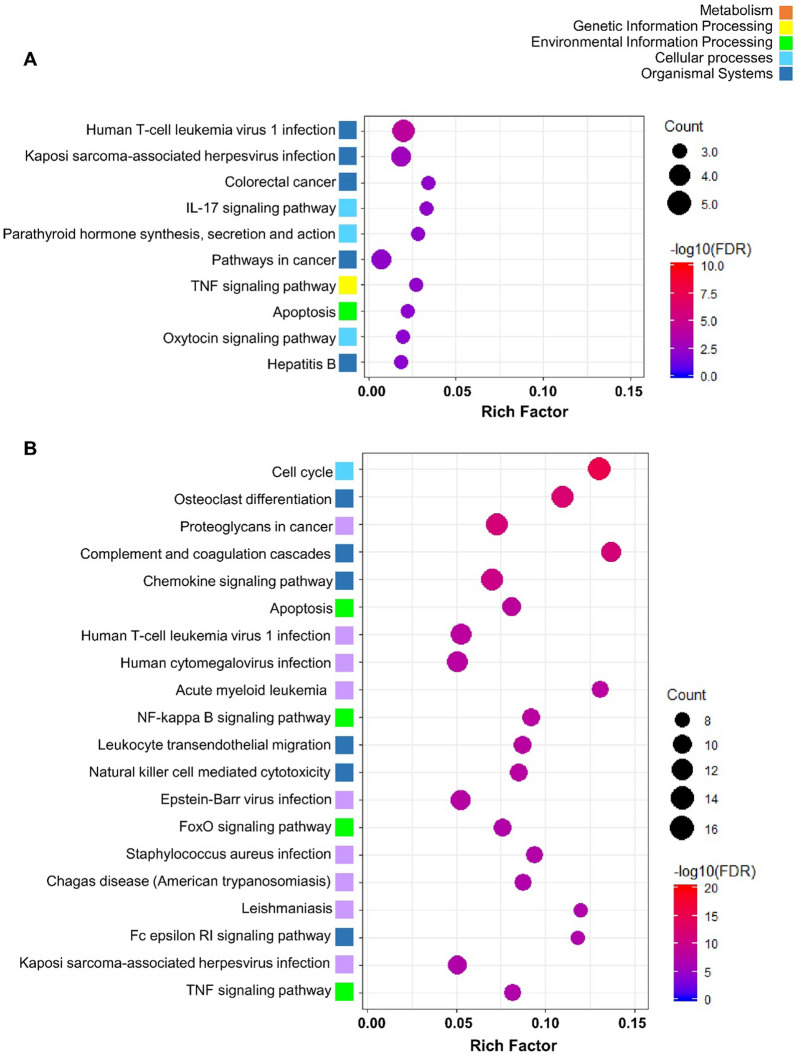

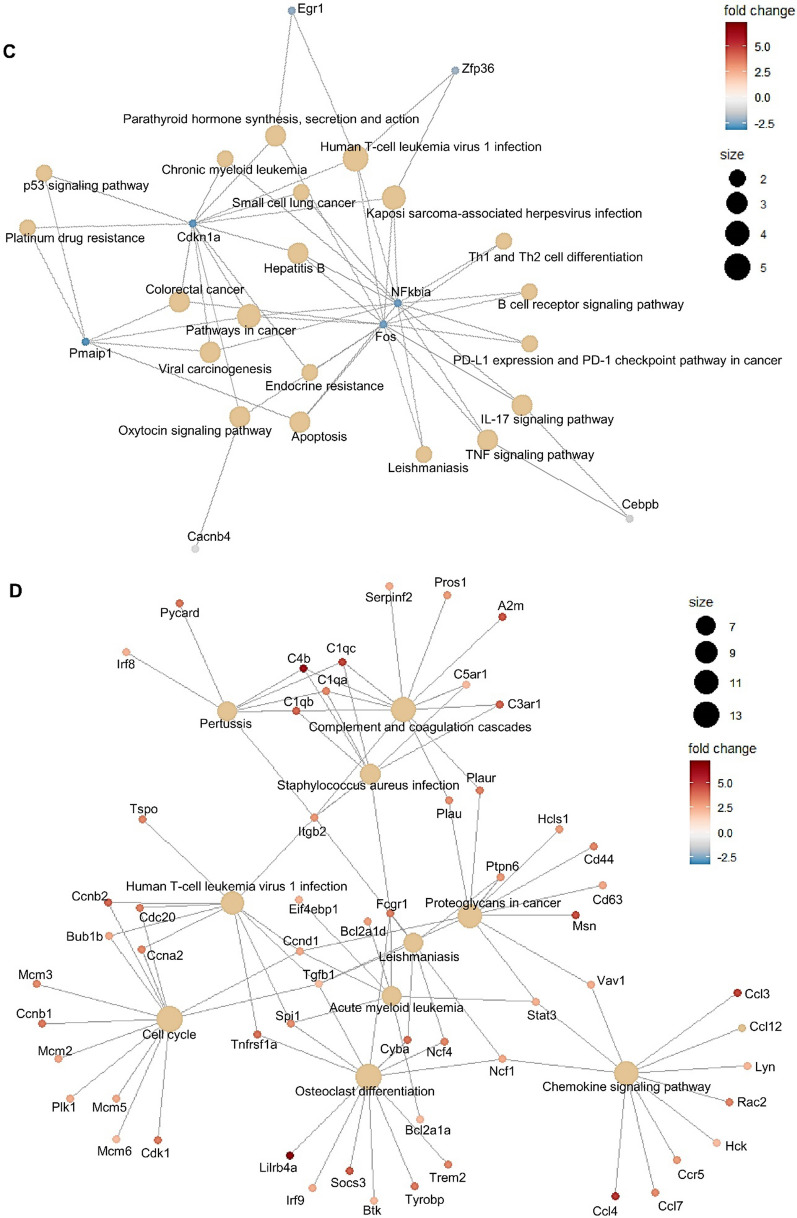
KEGG pathway enrichment analysis of DEGs in 0.25 MPa and 0.42 MPa. (**A**–**B**) KEGG pathway enrichment scatter plot of DEGs in the 0.25 MPa (**A**) and 0.42 MPa (**B**). The Y-axis shows the KEGG pathway of the top 10 KEGG enriched pathways (**A**) and the top 20 KEGG enriched pathways (**B**). The X-axis represents the rich factor, which was calculated by the ratio of the number of differentially expressed transcripts divided by the number of annotated transcripts in this pathway. This indicates the degree of KEGG pathway enrichment. The dot size and color represent the gene number and log_10_ FDR value, respectively. The low FDR values are in blue, and the high values are in red. (**C**–**D**) The interaction network of KEGG pathway enrichment analysis of hub genes at 0.25 MPa (**C**) and 0.42 MPa (**D**) at 48 h post-BBBD. The circle colors of genes represent expression levels. Red and blue colors indicate that the nodes are upregulated and downregulated, respectively. The area of each node indicates the degree of connectivity between the pathways and genes. The four downregulated hub genes were involved in the top 20 pathways (**C**). The 58 upregulated genes were involved in the top 10 pathways (**D**)

To further elucidate the biological functions and key pathways of all the DEGs in BBBD conditions with 0.25 and 0.42 MPa, we performed KEGG pathway enrichment analysis (Fig. 3). Significantly enriched pathways were identified among each group using a cutoff of FDR value < 0.05. In the present study, 24 DEGs had annotations that belonged to 12 KEGG pathways in the 0.25 MPa group, and 258 DEGs had annotations to 103 KEGG pathways in the 0.42 MPa group. The 10 most enriched pathways in the 0.25 MPa group are represented in Fig. 3a. The most significantly enriched KEGG pathway with annotation for each highly presented profile was “human T-cell leukemia virus 1 infection” belonging to the “organismal systems” category (Fig. 3a). At 48 h in 0.25 MPa condition, pathway interaction networks of the top 20 KEGG pathways were constructed with their associated 8 downregulated genes, as shown in Fig. 3c. The four major hub genes *Pmaip1*, *Cdkn1a*, *Fos*, and *NFκbia* intersected with several different pathways. The shared downregulated genes influence cytokine signaling during cell proliferation, viral infection, and carcinogenesis (Fig. 3c). The 20 most enriched KEGG pathways in the 0.42 MPa group are presented in Fig. 3b. The most significantly enriched KEGG pathway was the “cell cycle” belonging to the “cellular component” category (Fig. 3b). The top 10 KEGG pathway networks were constructed with 58 upregulated genes at 48 h in 0.42 MPa group (Fig. 3d). All associated genes were upregulated. The four most observed pathways (cell cycle, complement and coagulation cascades, osteoclast differentiation, and complement and coagulation cascades) were related to cell proliferation and inflammation (Fig. 3d). These data suggest that FUS-BBBD (0.42 MPa) promotes the increase of gene expression associated with inflammatory or cellular proliferation, resulting in several related pathways.

### The inflammatory response following FUS-BBBD related to two parameters

**Fig. 4:**
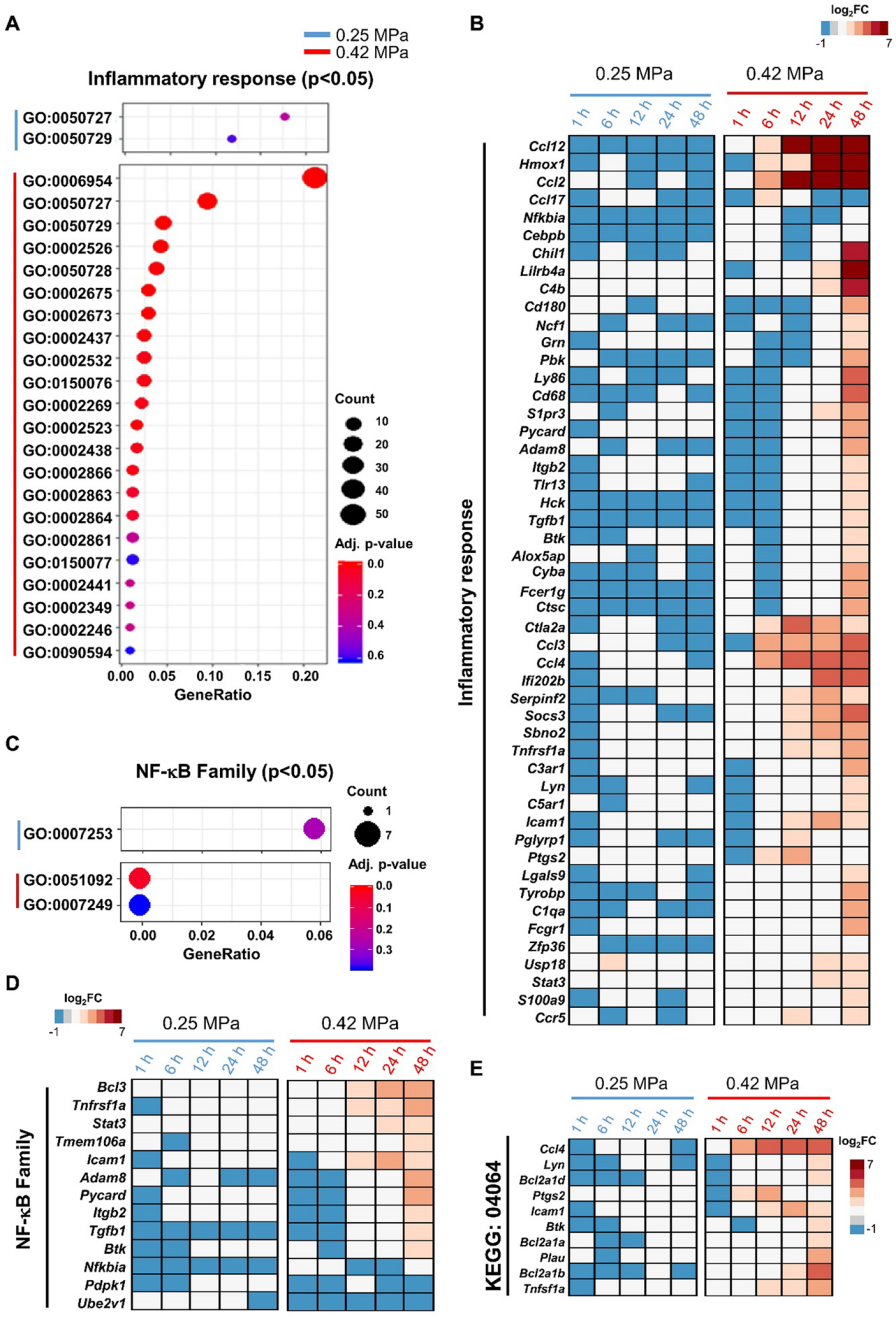

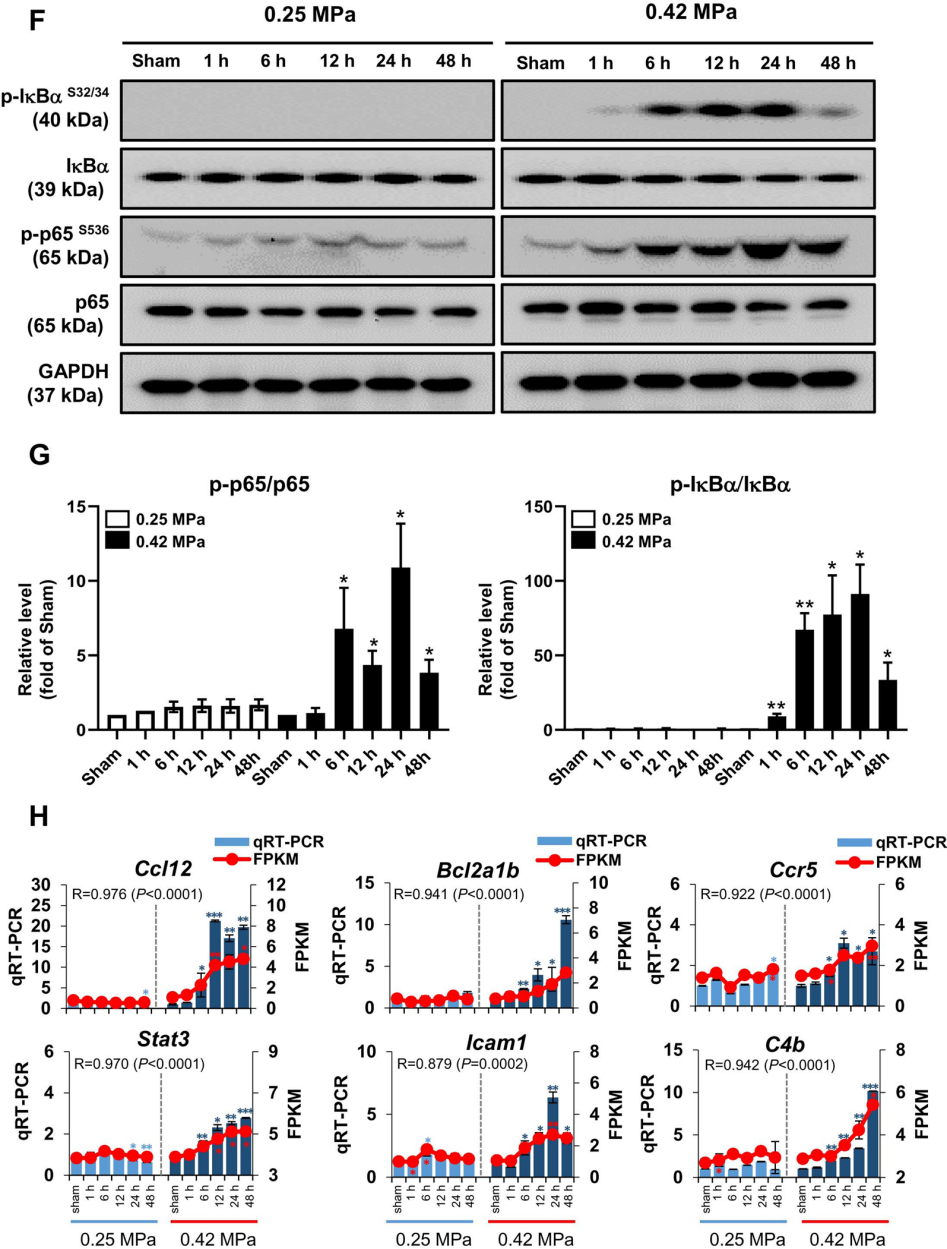
The molecular expression associated with the inflammatory response following FUS- BBBD. (**A**, **C**) Scatterplot analysis of the inflammatory response (**A**) and NF-κB pathway-related GO terms (**C**) of DEGs in the 0.25 MPa (blue line) and 0.42 MPa (red line) groups. Scale bar indicates significant enrichment terms (adjective *P* < 0.05). The circle size indicates the number of DEGs corresponding to the GO terms. (**B**, **D**) Heat map of genes associated with the inflammatory response (**B**) and NF-κB family-related GO terms (**D**) at 0.25 MPa (blue line) and 0.42 MPa (red line). (**E**) Heap map of the 10 genes corresponding to KEGG:04064 (NF-κB signaling pathway). The bar represents the scale of the expression levels for each gene (log_2_FC) in the color tape from low (blue) to high (red). (**F**) Evaluation of the phosphorylation status of IκBa (40 kDa) and p65 (65 kDa) protein expression was analyzed by western blot analysis in rat brain tissues at 1–48 h after BBBD. GAPDH was used as a loading control. (**G**) Densitometric analysis of western blot results presented in (**F**). Western blots were normalized to total protein and densitometric analysis was performed using image processing software. Values are mean ± standard error of the mean (SEM) in two independent experiments. (**H**) Verification of correlation between RNA-seq data and qRT-PCR of six representative transcripts. The light blue (0.25 MPa) and dark blue (0.42 MPa) columns represent the qRT-PCR results (left y-axis). The red lines indicate the FPKM values (right y-axis). The error bars show the standard deviation (STDEV) for the replicates in each experiment. *P < 0.05, **P < 0.01, ***P < 0.001 (two-tailed Student’s *t*-test; *vs.* Sham)

To better understand the inflammatory response after FUS-BBBD, we analyzed GO terms related to neuroinflammation. In the GO terms in 0.25 MPa group, “regulation of inflammatory response” (GO:0050727) and “positive regulation of inflammatory response” (GO:0050729) showed significant enrichment (*P* < 0.05) (Fig. 4a; Additional file 1: Table S3). 22 GO terms (including the same GO terms to 0.25 MPa) were observed in the 0.42 MPa group (Fig. 4a; Additional file 1: Table S4). The heat map analysis showed that the expression level of the DEGs associated with inflammation-related GO terms observed notably increased at the later time point in 0.42 MPa group, including genes associated with chemokines (*Ccl12*, *Ccl2*, *Ccl17*, *Ccl3*, *Ccl4*, and *Ccr5*), complement system (*C4b* and *C3ar1*, *C5ar1* and *C1qa*), immune cell activation (*Cd180*, *Cd68*, *Icam1*, and *Fcgr1*) (Fig. 4b). Concurrently, we analyzed GO terms related to the NF-κB signaling pathway. The GO term of ‘cytoplasmic sequestering of NF-κB’ (GO:0007253) was enriched in the 0.25 MPa group (Fig. 4c; Additional file 1: Table S5). In the 0.42 MPa group, the GO terms of ‘positive regulation of NF-κB transcription factor activity’ (GO:0051092) and ‘I-κB kinase/NF-κB signaling’(GO:0007249) were enriched (Fig. 4c; Additional file 1: Table S6). The expression of NF-κB pathway-associated genes belonging to GO terms 0007253, 0051092, and 0007249 were generally increased from 12 h later in the 0.42 MPa group (Fig. 4d). Furthermore, the ‘NF-κB signaling pathway’ (KEGG:04064) was significantly enriched among the KEGG categories associated with the inflammatory response (Fig. 4e). The expression levels of an inflammatory response or NF-κB pathway-related genes exhibited highly dynamic changes at the 0.42 MPa in a time-dependent manner (Fig. 4e). These DEGs trends indicate that the 0.42 MPa is a toxic FUS parameter that induces inflammation, NF-κB signaling, and immune cell infiltration.

Next, we determined whether the activation of NF-κB signaling pathway-related proteins was consistent with the results of transcriptome analysis (Fig. 4f). Phosphorylated expression of IkBα was markedly increased in the 0.42 MPa group from 6 h to 24 h post-BBBD. Furthermore, phospho-NF-κB p65, known as the downstream target of IκBa, was also markedly increased at the later time point (0.42 MPa) (Fig. 4f). To further evaluate the reliability of RNA sequencing analyses, the representative genes (*Ccl12*, *Bcl2a1b*, *Ccr5*, *Stat3*, *Icam1*, and *C4b*) were selected from the heat map of the ‘inflammatory response’ and analyzed by real-time RT-PCR (Fig. 4g). *Ccl12*, *Ccr5,* and *C4b* are inflammatory response-related GO terms; *Bcl2a1b* is assigned to KEGG04064 (NF-κB signaling pathway); and *Stat3* and *Icam1* belong to inflammatory response and NF-κB family related GO terms, respectively. The results demonstrated that the trend in the expression of DEGs was consistent with the real-time qRT-PCR results, confirming the reliability of the sequencing data (Fig. 4g). In summary, while the 0.42 MPa condition promoted FUS-BBBD-mediated inflammatory responses via NF-κB signaling, the 0.25 MPa condition of the biological response was negligible.

### Alteration of a subtype of reactive astrocytes by FUS-BBBD

**Fig. 5:**
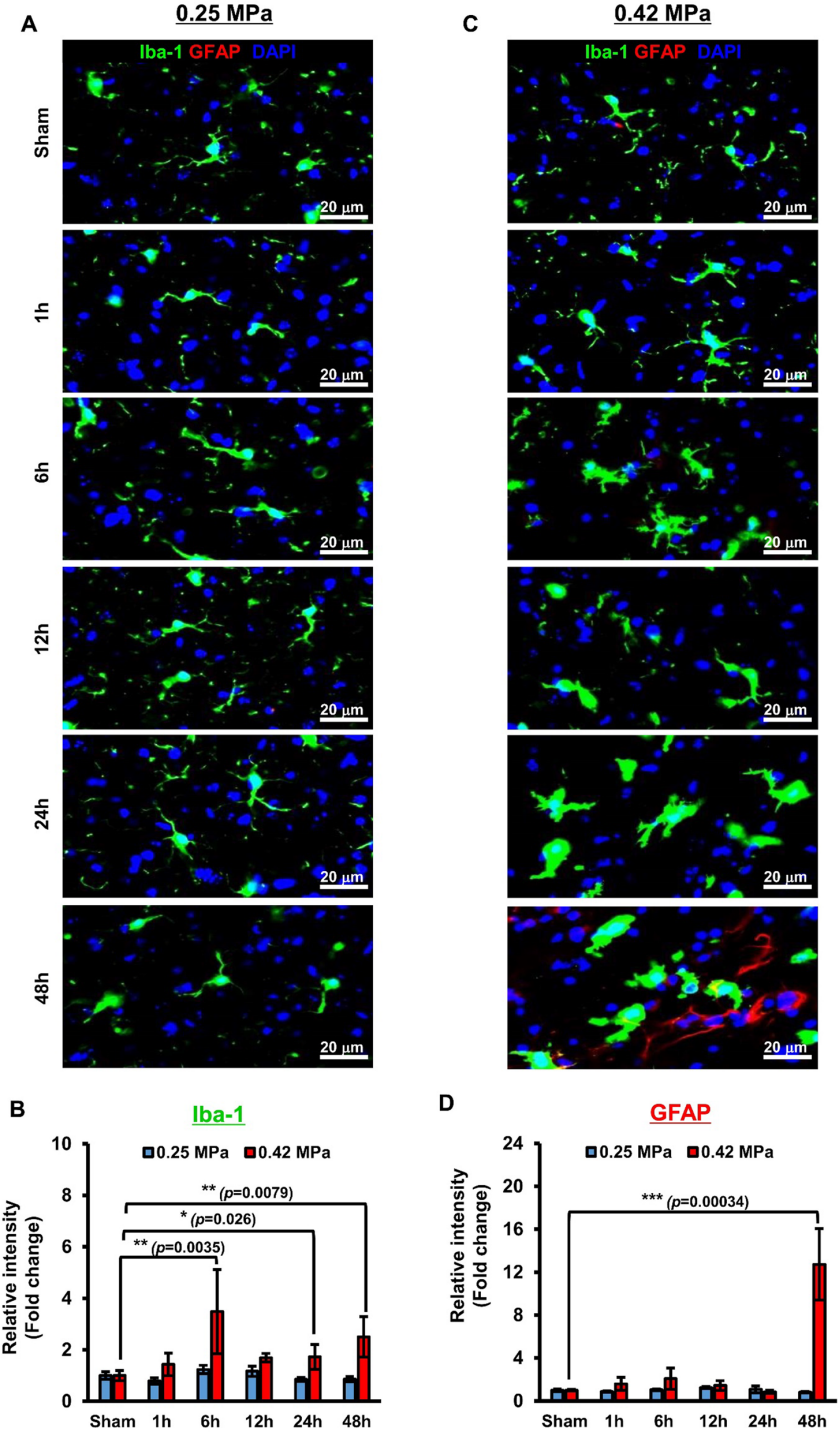
Histological evaluation of glia cell expression in the targeted brain tissues post-FUS BBBD. (**A**–**B**) Immunohistochemical analysis of glial cell expression. The brain thalamus sections were stained with Abs against Iba-1(microglia marker, green), GFAP (astrocyte marker, red), or DAPI (blue) at 1–48 h after differential FUS parameter-mediated BBBD in 0.25 MPa (**A**) or 0.42 MPa (**B**) (n = 2). (**C-D**) The bar graphs represent the fluorescence intensities for Iba-1 and GFAP. The values were calculated from each of the eight ROIs per hemisphere for the BBBD region (magnification, ×20). Data are presented as mean ± SD. *P < 0.05, **P < 0.01, ***P < 0.001 (two-tailed Student’s *t*-test; *vs.* Sham). (See Figure S5 for representative whole-brain sections stained for Iba-1 and GFAP)

We used immunofluorescence (IF) to determine glial cell expression patterns and morphology in the brain tissues following FUS-BBBD in a time-dependent manner (Additional file 1: Figure S6). As expected, significant activation was observed in Iba-1^+^ cells between 1 and 48 h post-BBBD at 0.42 MPa compared to the sham control (Fig. 5a, b). Furthermore, the fluorescence intensity of GFAP^+^ cells was significantly increased at 48 h after the 0.42 MPa FUS parameter compared with the sham control (Fig. 5c, d). In response to the 0.25 MPa FUS parameter, glial cells (Iba-1^+^ and GFAP^+^) were not significantly altered in the BBBD region. These findings showed that robust induction of reactive glial cells was increased in the 0.42 MPa group, while the 0.25 MPa group had sufficient acoustic pressure to open the BBB without neuroglial activation, and thus, did not induce inflammation.

**Fig. 6:**
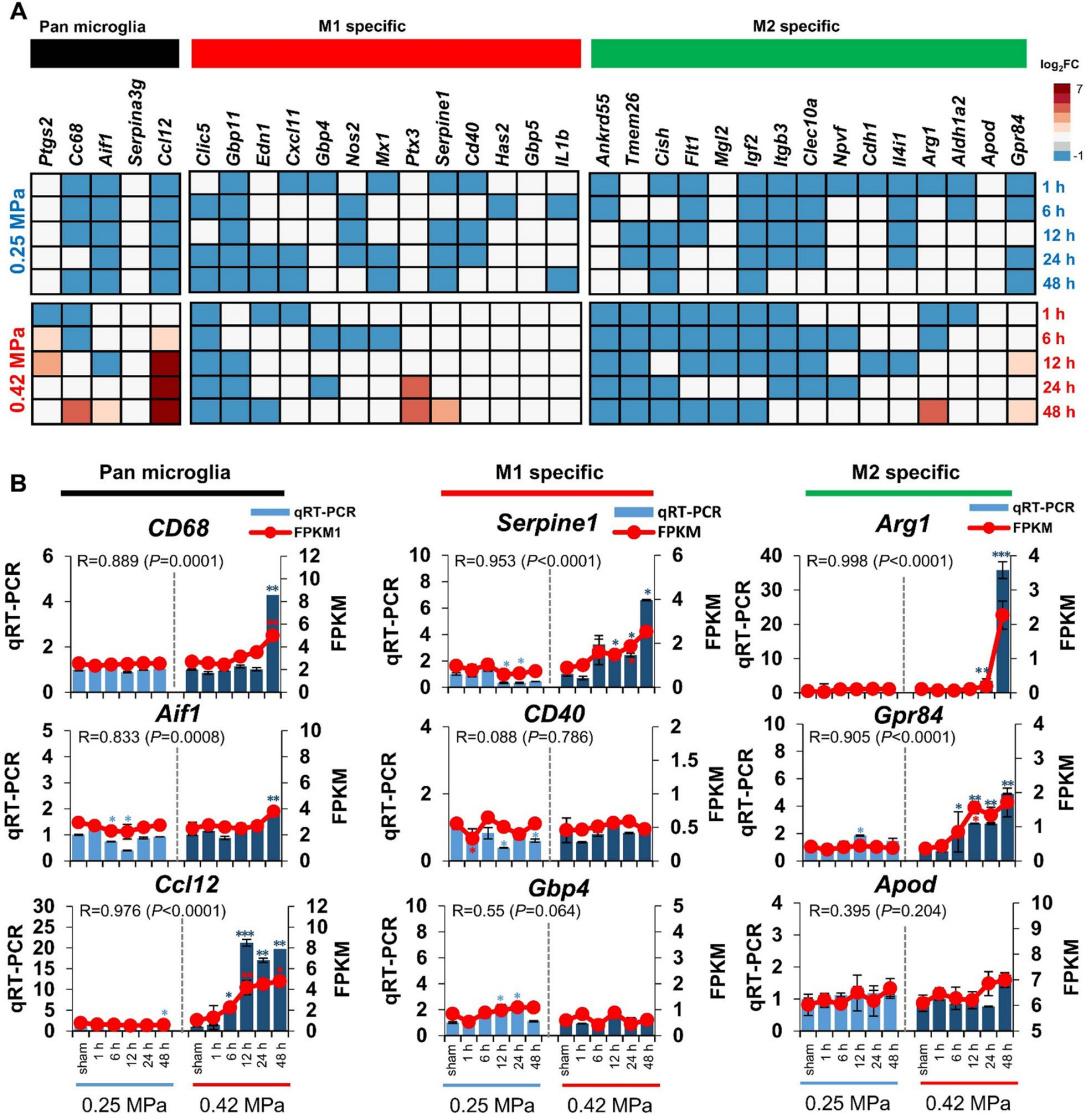
Differential gene expression analysis of subtype of microglia in brain tissue post-FUS BBBD. (**A**) Heat map of microglia-specific transcripts. The heat map showing the clustering of M1 and M2 genes in response to FUS-BBBD (0.25 MPa and 0.42 MPa). The DEGs based on log_2_FC values were divided into pan microglia (black), M1-specific (red), and M2-specific (green) transcripts in the RNA sequence data set. Blue and red indicate a decrease and increase in expression compared with the sham control, respectively. (**B**) Gene expression correlation between qRT-PCR and RNA-seq data *CD68*, *Aif1*, and *Ccl12* are pan-microglia-specific genes. *Sepine1*, *CD40*, and *Gbp4* are M1-specific genes. *Arg1*, *Gpr84*, and *Apod* are M2-specific genes. The light blue (0.25 MPa) and dark blue (0.42 MPa) columns represent the qRT-PCR results (left y-axis). The red lines indicate the FPKM values (right y-axis). The error bars show the standard deviation (STDEV) for the replicates in each experiment. *P < 0.05, **P < 0.01, ***P < 0.001 (two-tailed Student’s *t*-test; *vs.* Sham)

**Fig. 7:**
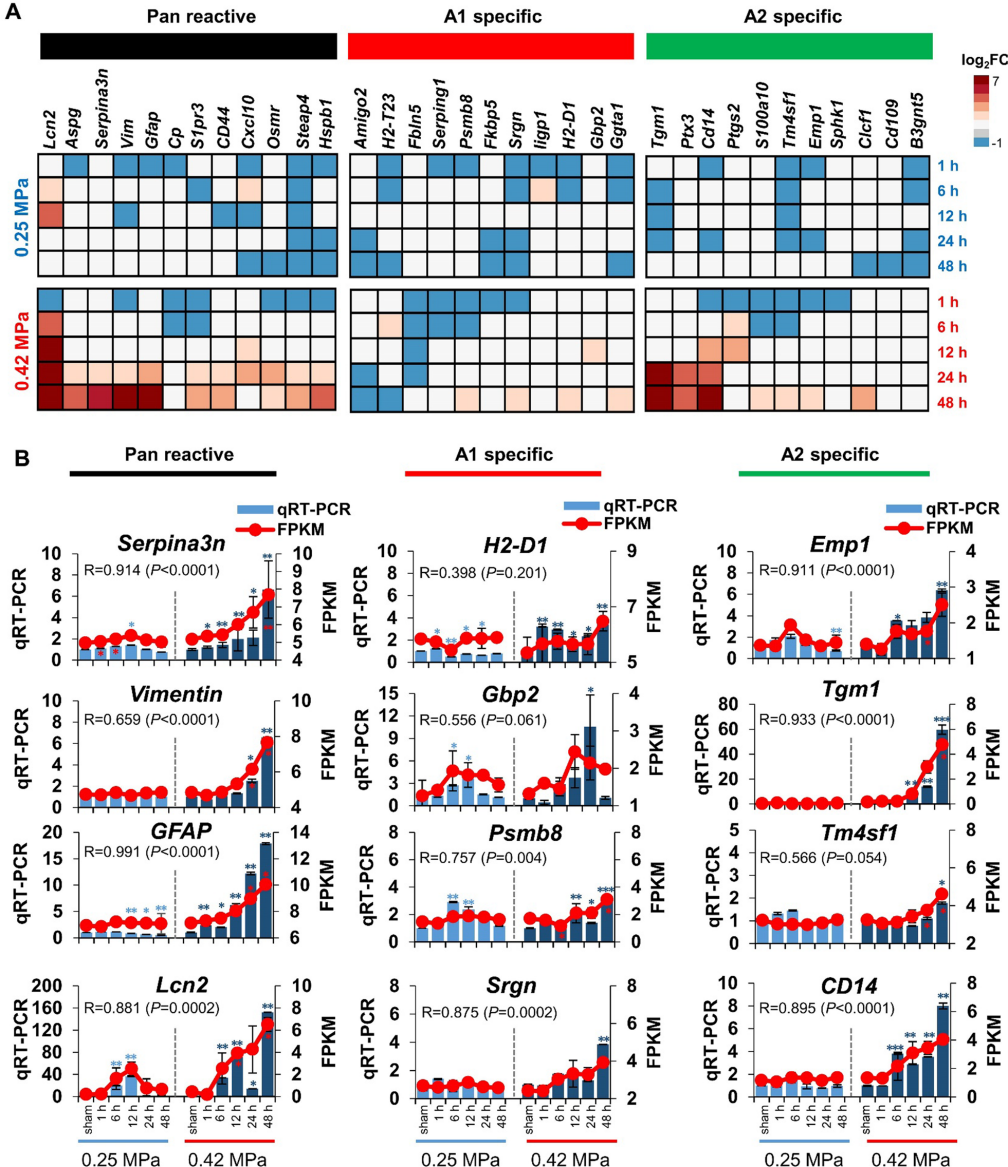
Differential gene expression analysis of subtype of astrocyte in brain tissue post-FUS BBBD. (**A**) Heat maps depicting log2FC values for astrocyte reactivity-specific marker genes, in response to FUS-BBBD (0.25 MPa and 0.42 MPa). The DEGs were divided into pan-reactive (black), A1-specific (red), and A2-specific (green) transcripts in the RNA-seq dataset. Blue and red indicate a decrease and increase in expression compared with the sham control, respectively. (**B**) Gene expression correlation between qRT-PCR and RNA-seq data *Serpina3n*, *vimentin*, *GFAP*, and *Lcn2* are pan-astrocyte-specific genes. *H2-D1*, *Gbp2*, *Psmb8*, and *Srgn* are A1-specific genes. *Emp1*, *Tgm1*, *Tm4sf1*, and *CD14* are A2-specific genes. The blue columns represent the qRT-PCR results (left y-axis). The red lines indicate the FPKM values (right y-axis). The error bars show the standard deviation (STDEV) for the replicates in each experiment. *P < 0.05, **P < 0.01, ***P < 0.001 (two-tailed Student’s *t*-test; *vs.* Sham)

We then investigated whether the two FUS conditions induced a change in glial cell subtype expression and found that RNA sequencing profiles were enriched in microglia/astrocyte-subtype-specific genes (Figs. 6, 7). Microglia-associated gene expression was not altered in the 0.25 MPa condition. Furthermore, although the pan microglia-associated genes were increased in the 0.42 MPa group between 6 and 48 h, there was could not be clearly polarized towards an M1 and M2 subtype (Fig. 6a) In addition, we found a significant correlation between qRT-PCR and FPKM values of representative three transcripts for each category of microglia (Fig. 6b). Overall expression patterns of transcripts for *CD40*, *Gbp4*, and *Apod* have seemed to be similar between qRT-PCR and FPKM, whereas there was no significant correlation. Next, we found that the pan reactive astrocyte-specific genes were upregulated in the 0.42 MPa group between 6 and 48 h. Furthermore, A2-specific astrocyte gene expression was higher than A1-specific genes in the 0.42 MPa group between 12 and 48 h (Fig. 7a). The expression patterns of reactive astrocyte phenotype-specific genes were significantly correlated with RNA sequencing data and real-time PCR (Fig. 7b). Despite the similar patterns of expression, there was no significant correlation between qRT-PCR and FPKM values for transcripts of *H2-D1*, *Gbp2* and *Tm4sf1*. The polarization of A2-astrocytes was a prominent at 0.42 MPa FUS-BBBD, and it suggested that the protective astrocyte, known as A2-type, was induced in response to the massive FUS-BBBD, leading to brain damage.

## Discussion

This study investigated the safety of FUS-induced BBB permeability, via histopathological and transcriptome analyses, under two different sonication conditions, 0.25 MPa and 0.42 MPa, which induced distinct BBB permeability and biological responses. In this study, we found that the BBB was successfully disrupted at a 0.25 MPa sonication without neuroinflammation or neurodegeneration. However, the 0.42 MPa sonication resulted in significant upregulation of NF-κB signaling pathway-associated molecules, and activation of microglia and astrocytes, which is similar to previous studies [13, 26, 28]. Intriguingly, activated astrocytes were predominantly of the A2, anti-inflammatory phenotype. This finding implies that although the 0.42 MPa FUS condition induced inflammation, it could lead to tissue repair in the brain through A2-type reactive astrocytes. To our knowledge, this is the first report to suggest the functional role of neuroglial cells in resolving inflammation events, which are the biological responses post-BBBD.

The acoustic pressure amplitude is important for determining the BBB opening efficacy and tissue damage by affecting the oscillation of microbubbles circulating in the vasculature [54]. We applied two different acoustic pressures to investigate the safety of FUS-BBBD. We selected 0.25 MPa sonication condition as the sufficient FUS parameter to validate the biological safety post-BBBD because this sonication condition was widely used in the mouse brain to disrupt the BBB in several studies safely [55, 56]. In previous pre-clinical studies [57, 58], a drug such as doxorubicin was successfully delivered after FUSinduced permeability changes with a *K*_*trans*_ range from 0.0086 min^−1^ to 0.0232 min ^−1^. In this study, the permeability properties under 0.25 MPa of sonication revealed a mean *K*_*trans*_ of 0.02 ± 0.005 min^−1^, implying that it ensures efficacy for drug delivery (Fig. 1d). Furthermore, the histological findings revealed no significant difference compared to the contralateral region, demonstrating the feasibility of a safe and efficient FUS-mediated BBBD under 0.25 MPa of acoustic pressure (Fig. 1g–i). We applied a higher sonication condition (0.42 MPa) to compare the difference according to the extent of BBBD. As the pressure amplitude increased, the BBB permeability increased compared to 0.25 MPa (Fig. 1a–d), but such observations were accompanied by visible microhemorrhage, microvacuolation, and neurodegeneration (Fig. 1g–i), which is consistent with previous reports [54, 59].

The differences in permeability and damage are closely related to the type of MB activity that results from FUS exposure [56]. Thus, we elucidated the interaction between MB activity and local damage during BBB permeabilizing by monitoring the acoustic emissions. The 0.25 MPa acoustic pressure mainly induced stable cavitation (i.e., harmonics) that may cause a stable BBB opening without visible damage (Fig. 1e–i). On the other hand, 0.42 MPa generated harmonics and ultra-harmonics with broadband emissions, indicating inertial cavitation associated with undesirable damage (Fig. 1e–i). These results are consistent because inertial cavitation, as detected by wideband acoustic emission in vivo, is likely to be a major factor in damage [60]. Also, our results suggest that a significant increase in ultra-harmonic emission might lead to neuronal damage during excessive FUS exposure (Fig. 1f, g–i). Recent evidence indicates that the ultra-harmonic components could be a relevant indicator for damage prediction apart from inertial cavitation [23], and our results support this finding. Differences in microbubble doses, size distribution, and injection methods can result in differences in BBB permeability and tissue damage, even when using the same FUS-BBBD procedure. Indeed, several studies have demonstrated different MB dose-dependent BBB opening levels and bioeffects [32, 61]. In this study, we chose one condition of the Definity dose to simplify the experimental conditions and reduce the variation in the MB condition when the FUS parameters were applied. The optimal MB dose to elicit a sterile inflammatory response following FUS-BBBD is still controversial. Kovacs et al. suggested that compared with a dose of 10 µl/kg [32], a Definity dose > 20 μL/kg will cause an inflammatory response after FUS-BBBD. Furthermore, several reports demonstrated that FUS-BBBD combined with Definity at a higher dose (20–80 μL/kg) could induce opening of the BBB and biological effects. Therefore, a Definity dose of 20 μL/kg was selected to open the BBB.

Several studies have raised concerns over the safety issues associated with the inflammatory response to FUS-BBBD [13, 26, 28, 62]. However, the evaluation of these biological responses has been limited to superficial changes such as histological damage, hemorrhage, glial cell activation, and the microvascular transcriptome under a single FUS parameter time interval. It is difficult to comprehensively investigate the biological effects on the microenvironment of the BBBD region. Therefore, to correlate biological responses with ultrasound acoustic pressure amplitude to determine safety, we performed genome-wide transcriptome analyses to observe alterations in the brain’s molecular event in BBBD regions treated with FUS in a time-dependent manner. Our results showed scarce changes in genomic expression (0.14%) in the 0.25 MPa sonicated tissue, and DEGs were slightly downregulated (Fig. 2c) compared to 0.42 MPa sonication. Interestingly, the hub genes *FOS* and *EGR1* categorized from KEGG pathway enrichment analysis were decreased after BBBD induced by 0.25 MPa exposure (Fig. 3c). The *FOS* and *EGR1* genes belonging to the immediate-early gene (IEG) family have been reported to be induced by a secondary insult following brain injuries [63, 64]. We also found no significant changes in genes and proteins involved in inflammation through the NF-kB signaling pathway until 48 h post-BBBD in 0.25 MPa sonicated tissue (Fig. 4). Although further detailed molecular basis for these findings remains to be determined, these results indicate that the 0.25 MPa condition is not involved in the acute phase inflammatory response. Our results suggest that FUS-BBBD is distinct from the damage-induced BBB dysfunction, although the optimal FUS parameter (0.25 MPa) permeabilizes the BBB sufficiently.

On the other hand, the number of DEGs (1.47%) in 0.42 MPa sonicated tissue was greater than 0.25 MPa and upregulated in a time-dependent manner (Fig. 2d). We found that 0.42 MPa upregulated chemokine (*CCL12, CCL2, CCL17, CCL3, CCL4,* and *CCR5*), endothelial adhesion molecule (*ICAM1*), complementary response (*C4b, C3ar1, C5ar1, C1qa*), and immune cell activation genes (*CD180, CD68, FCGR1*) between 6 and 48 h post-BBBD (Fig. 4b). Furthermore, NF-κB pathway-associated genes expression was increased under 0.42 MPa sonication condition compared to 0.25 MPa sonication condition (Fig. 4d, e). These results were consistent with previous studies, demonstrating that excessive FUS-BBBD induced sterile neuroinflammation via the NF-κB pathway [26, 28, 62]. In general, the brain endothelium immediately releases cytokines and chemokines after detecting stimuli to recruit and activate platelets and leukocytes [65] that induce inflammation in the brain [66], as well as for transcription of genes to repair damaged tissues and blood vessels. Transient acute inflammation is important not only in the neuro-inflammation response but also in regeneration and neuroprotection [67]. Given the observed elevation of the pro-inflammatory gene expression at 0.42 MPa in the present study, it is important to consider that the stress-induced by 0.42 MPa FUS-BBBD could promote protective immunoreactivity.

Previous studies have shown that the activation of microglia and astrocytes could have different subtypes, both beneficial and detrimental, depending on the brain injury’s reactive status or disease [38, 68]. Despite the increasing research about glial cell phenotype activation after FUS-BBBD [13, 28], evidence for glial cell heterogeneity is not well established. To our knowledge, this study is the first to show the functional subtype shifts of reactive glial cells triggered by FUS-BBBD using gene profiling [38, 69]. We observed that alterations in morphology and differential gene expression in glial cells did not occur in 0.25 MPa-treated tissue for 48 h (Figs. 5, 6, 7). The 0.42 MPa FUS-BBBD elicited the apparent activation of microglia (from 1 to 48 h) and astrocytes (at 48 h) post-BBBD based on morphological changes (Fig. 5). In the present study, it was difficult to clearly distinguish the M1-/M2-subtype classification based on the genetic profile, despite the increased expression of pan-microglia marker genes (Fig. 6).

In contrast to microglia, A2-type astrocytes were more predominant than A1-type astrocytes in the 0.42 MPa FUS-BBBD condition (Fig. 7). Our results imply that the 0.42 MPa FUS-BBBD could affect A2-type astrocyte polarization, promoting brain recovery and repair. These results provide insight into a novel mechanism in which the reactive neuroglia leads to protective effects such as tissue homeostasis and attenuating inflammation by increasing A2-type astrocytes, despite inducing neuroinflammation.

Our study had some limitations; the findings are limited to an inflammatory response induced by FUS-BBBD in a healthy animal model. Further studies are needed in a disease model to understand the pathophysiological roles of glial cell polarization following FUS-BBBD. Next, the safety profiles were assessed for 48 h post-BBBD. A slight increase of A1-specific astrocyte genes was observed in the 0.42 MPa group at 48 h. Because of the temporary changes or reversible for the functional subtype shifts of reactive glial cells, it is important to understand the timing and complexity of the immune responses to translate the clinical outcome [70]. Thus, future studies should focus on characterizing the long-term impact of FUS-BBBD on brain tissues. Moreover, our data included only information on gene expression and phenotypic changes. To further define the effective role of A2-type astrocytes following FUS-mediated disruption, more detailed evidence of the mechanisms and pathways by which reactive A2 astrocytes are activated is required. We plan to address the functional roles of A2-type astrocytes in further work.

## Conclusions

Here, we identified the biological responses to BBBD, induced by different acoustic sonication pressures. Our findings suggest that sufficient BBBD conditions could be safe without vascular/tissue damage or sterile inflammatory responses in brain tissue. Although excessive sonication induces inflammation, it could lead to tissue repair and brain homeostasis through A2-type reactive astrocytes.

## Supplementary Information

**Additional file 1: Figure S1.** Experimental setup of the MRgFUS system. **Figure S2.** Contrast-enhanced T1-weighted MR image of a mouse brain after sonication. **Figure S3.** The representative whole brain section image was stained with H&E and FJC. **Figure S4.** Functional enrichment analysis of highly regulated differentially expressed genes (DEGs) in 0.25 MPa condition. **Figure S5.** Functional enrichment analysis of highly regulated differentially expressed genes (DEGs) in 0.42 MPa condition. **Figure S6.** Melting curve analysis with specificity of RT-qPCR amplification. **Figure S7.** The represented whole-brain sections for IF analysis of Iba-1 and GFAP post-BBBD. **Table S1.** The differential expressed genes in the 0.25 MPa condition. **Table S2.** The differential expressed genes in the 0.42 MPa condition. **Table S3.** GO categories associated with the inflammatory response in the 0.25 MPa. **Table S4.** GO categories associated with the inflammatory response in the 0.42 MPa. **Table S5.** GO categories associated with the NF-κB pathway in the 0.25 MPa. **Table S6.** GO categories associated with the NF-κB pathway in the 0.42 MPa. **Table S7.** Primers used for real-time qRT-PCR. **Table S8.** The MR parameters used in the study.

## Abbreviations

AD: Alzheimer’s disease
BBB: Blood–brain barrier
BBBD: Blood–brain barrier disruption
CNS: Central nervous system
DEGs: Differentially expressed genes
FDA: Food and drug administration
FDR: False discovery rate
FJC: Fluoro-jade C
FPKM: Fragments per kilobase of transcript per million
Gd-DTPA: Gadolinium-diethylenetriamine penta-acetic acid
GO: Gene ontology
ICD: Inertial cavitation dose
IgM: Immunoglobulin M
KEGG: Kyoto encyclopedia of genes and genomes
FUS: Focused ultrasound
MB: Microbubble
MRI: Magnetic resonance image
NHPs: Non-human primates
PCD PD: passive cavitation detector Parkinson’s disease
RBCs: Red blood cells
PRF: Pulse repetition frequency
RT-PCR: Reverse transcription-polymerase chain reaction
SCD_h_: Stable cavitation dose with harmonics
SCD_u_: Stable cavitation dose with ultraharmonics

## Declarations

### Data availability

The data presented in this study are available on request from the corresponding author.
