# Supplementary material for: The new insight into the inflammatory response following focused ultrasound-mediated blood–brain barrier disruption
Source: Fluids Barriers CNS. 2022 Dec 23;19:103. doi: 10.1186/s12987-022-00402-3 (PMC9783406; doi:10.1186/s12987-022-00402-3)
Supplement: Supplementary file 1 — Additional file 1: Figure S1. Experimental setup of the MRgFUS system. Figure S2. Contrast-enhanced T1-weighted MR image of a mouse brain after sonication. Figure S3. The representative whole brain section image was stained with H&E and FJC. Figure S4. Functional enrichment analysis of highly regulated differentially expressed genes (DEGs) in 0.25 MPa condition. Figure S5. Functional enrichment analysis of highly regulated differentially expressed genes (DEGs) in 0.42 MPa condition. Figure S6. Melting curve analysis with specificity of RT-qPCR amplification. Figure S7. The represented whole-brain sections for IF analysis of Iba-1 and GFAP post-BBBD. Table S1. The differential expressed genes in the 0.25 MPa condition. Table S2. The differential expressed genes in the 0.42 MPa condition. Table S3. GO categories associated with the inflammatory response in the 0.25 MPa. Table S4. GO categories associated with the inflammatory response in the 0.42 MPa. Table S5. GO categories associated with the NF-κB pathway in the 0.25 MPa. Table S6. GO categories associated with the NF-κB pathway in the 0.42 MPa. Table S7. Primers used for real-time qRT-PCR. Table S8. The MR parameters used in the study. [file 12987_2022_402_MOESM1_ESM.doc]

**Additional file information**

**THE NEW INSIGHT INTO THE INFLAMMATORY RESPONSE FOLLOWING FOCUSED ULTRASOUND-MEDIATED BLOOD-BRAIN BARRIER DISRUPTION**

Hyo Jin Choi^1^,, Mun Han^1^, Hyeon Seo^2^, Chan Yuk Park^1^, Eun-Hee Lee^1,*^, and Juyoung Park^3,*^

*^1^Medical Device Development Center, Daegu-Gyeongbuk Medical Innovation Foundation (K-MEDI Hub), 80, Cheombok-ro, Dong-gu, Daegu, 41061, Republic of Korea*

*^2^Department of Computer Science, Gyeongsang National University, 501, Jinju-daero, Jinju-si, Gyeongsangnam-do, 52828, Republic of Korea*

*^3^College of Future Industry, Department of High-tech Medical Device, Gachon University, 1342, Seongnam-daero, Sujeong-gu, Seongnam-si, Gyeonggi Province, 13120, Republic of Korea*

**Corresponding author:*

*Eun-Hee Lee*

*Medical Device Development Center, Daegu-Gyeongbuk Medical Innovation Foundation (K-MEDI hub), 80 Cheombok-ro, Dong-gu, Daegu, 41061, Republic of Korea.*

*Tel: +82-53-790-5597; Fax: +82 053-790-5519; E-mail address:* [*ehlee@kmedihub.re.kr*](mailto:ehlee@kmedihub.re.kr)

*Juyoung Park*

*College of Future Industry, Department of High-tech Medical Device, Gachon University, 1342, Seongnam-daero, Sujeong-gu, Seongnam-si, Gyeonggi Province, 13120, Republic of Korea*

*Tel: +82-31-750-2640; Fax: +82-31-750-2631; E-mail address: opedoors@gachon.ac.kr*

**This file includes:**

**Additional file 1: Figures S1 to S7**

**Additional file 1: Tables S1 to S8**

**
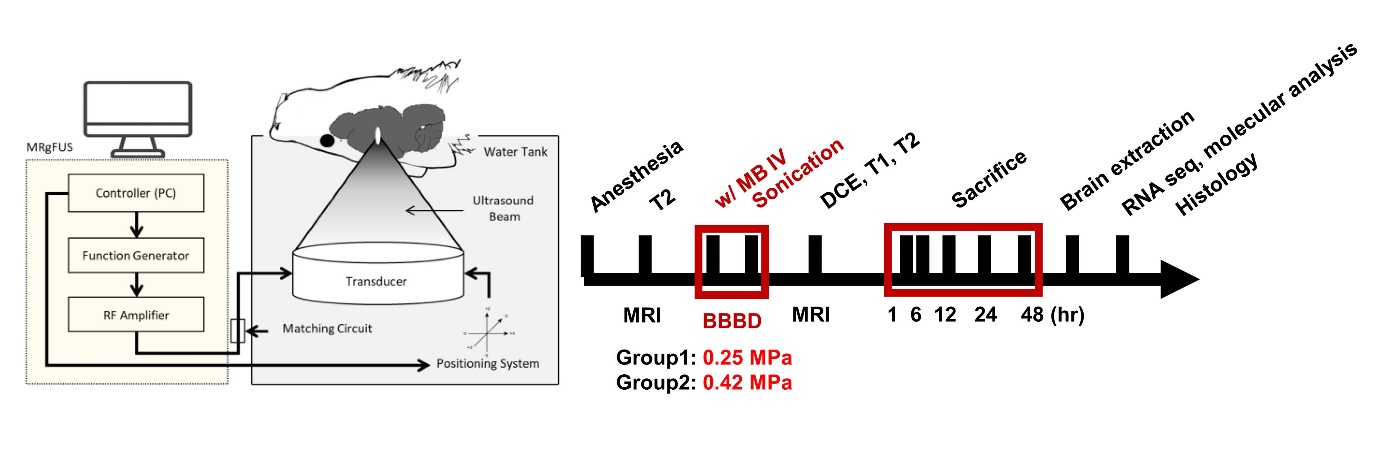
**

**Additional file 1: Figure S1. Experimental setup of the MRgFUS system**

Schematic illustration of MRgFUS systems for BBBD in a mouse model (left panel). Experimental schedule for each experimental purpose (right panel)

**
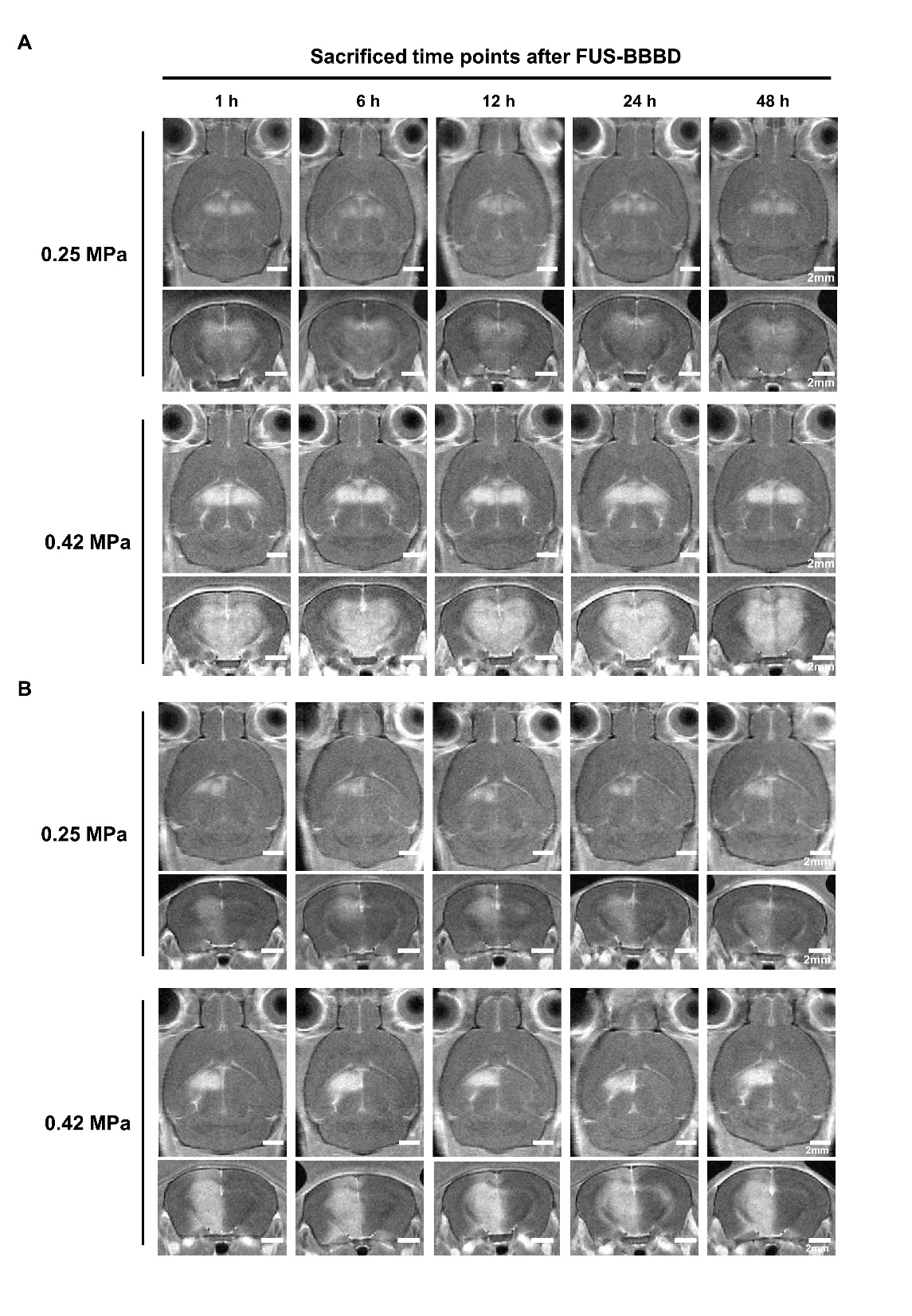
**

**Additional file 1: Figure S2. Contrast-enhanced T1-weighted MR image of a mouse brain after sonication**

**a.** For the RNA seq and molecular-based experiments, the thalamus region’s four points were sonicated with different FUS parameters. The upper and bottom panel showed 0.25 MPa and 0.42 MPa, respectively. (n = 2 per time point) **b.** For immunohistochemistry, the two points of the thalamus region in the hemisphere were sonicated with different FUS parameters (n = 2, per time point). The coronal and axial slices of a mouse brain in vivo, 1, 6, 12, 24, and 48 h after FUS BBBD, are presented. Following each FUS parameter, the extent of BBB opening showed a certain and adequate level. Scale bar, 2 mm.

**
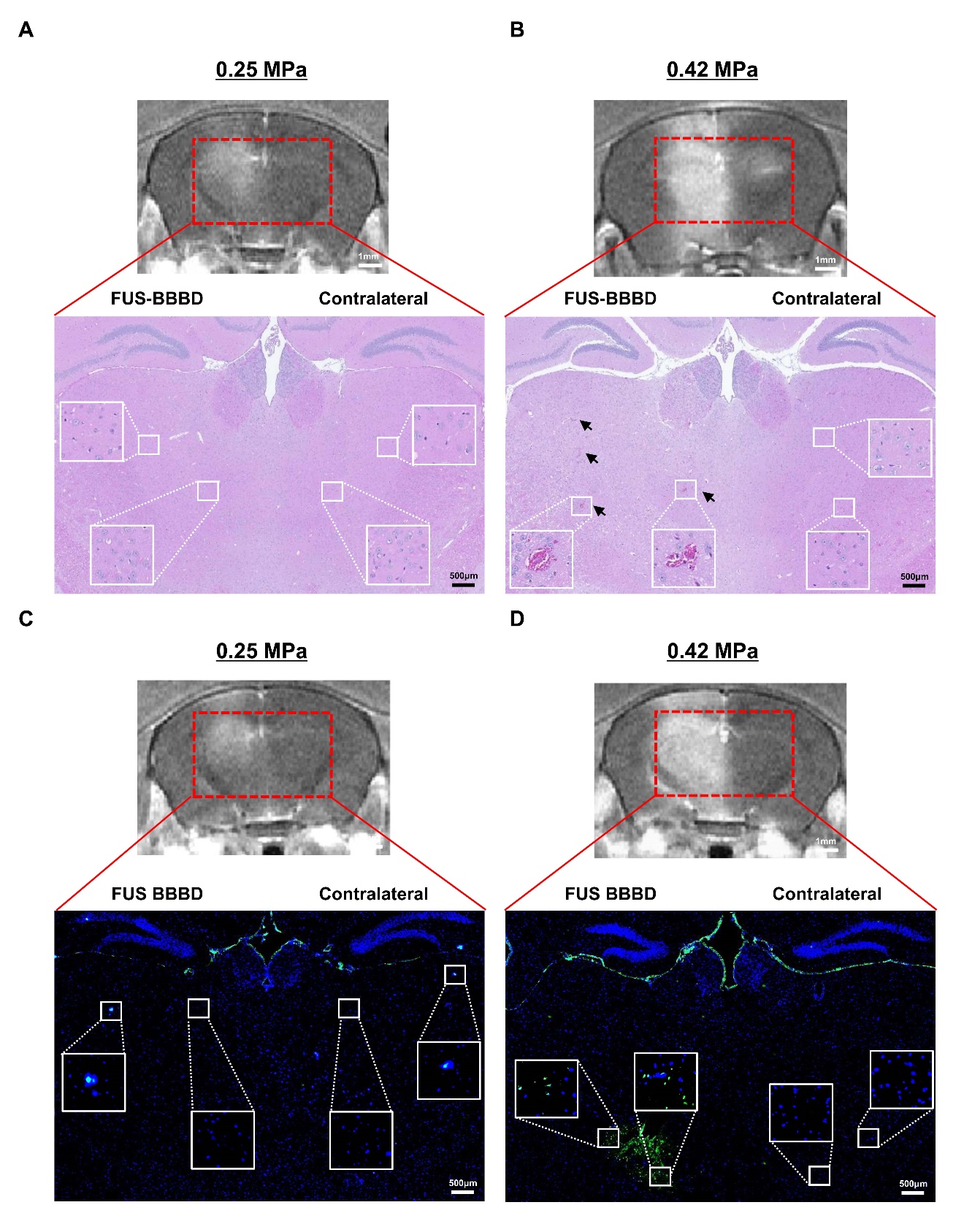
**

**Additional file 1: Figure S3. The representative whole brain section image was stained with H&E and FJC**

**a, b.** Brain sections were stained with H & E staining for histology to confirm the micro-hemorrhage according to the FUS parameter-mediated BBBD. The mice were sacrificed 4 h post-BBBD (n = 2). Compared to the MR image for the BBBD region (dashed red line), histological analysis was performed. At 0.25 MPa, micro-hemorrhages were not detected in selected regions (white squared box) of H & E images between the contralateral and FUS-BBBD hemispheres (a). Multiple extravasated red blood cells (RBC) (black arrows and enlarged image, white squared box) were detected in the FUS-BBBD parenchyma region at 0.42 MPa (b) **c, d.** Representative whole brain sections were stained with FJC to detect neuronal damages (scale bar, 500 μm). The mice were sacrificed after 24 h BBBD (n = 2). FJC-positive neuronal cell death was observed in the immunofluorescence section of the FUS-BBBD region. The white square box images were magnified 20 times (original magnification, x20).

**
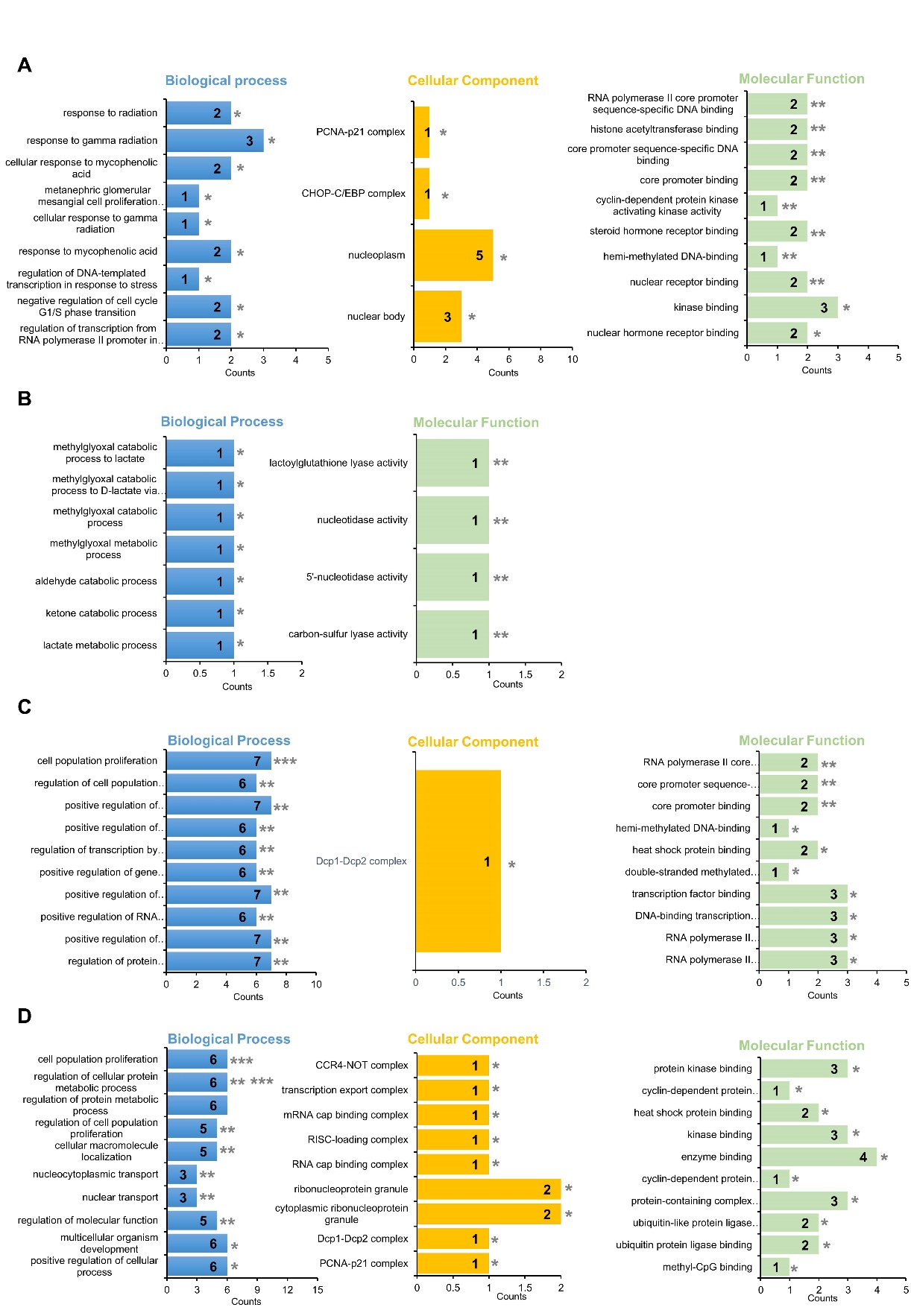
**

**Additional file 1: Figure S4. Functional enrichment analysis of highly regulated differentially expressed genes (DEGs) in 0.25 MPa condition**

GO functional analysis of DEGs at 6 h (**a**), 12 h (**b**), 24 h (**c**), and 48 h (**d**) compared with the sham control. They were annotated using three ontology categories: biological process (blue), cellular component (yellow), and molecular function (green). (**p* < 0.05, ***p* < 0.01 and, ***p < 0.001).


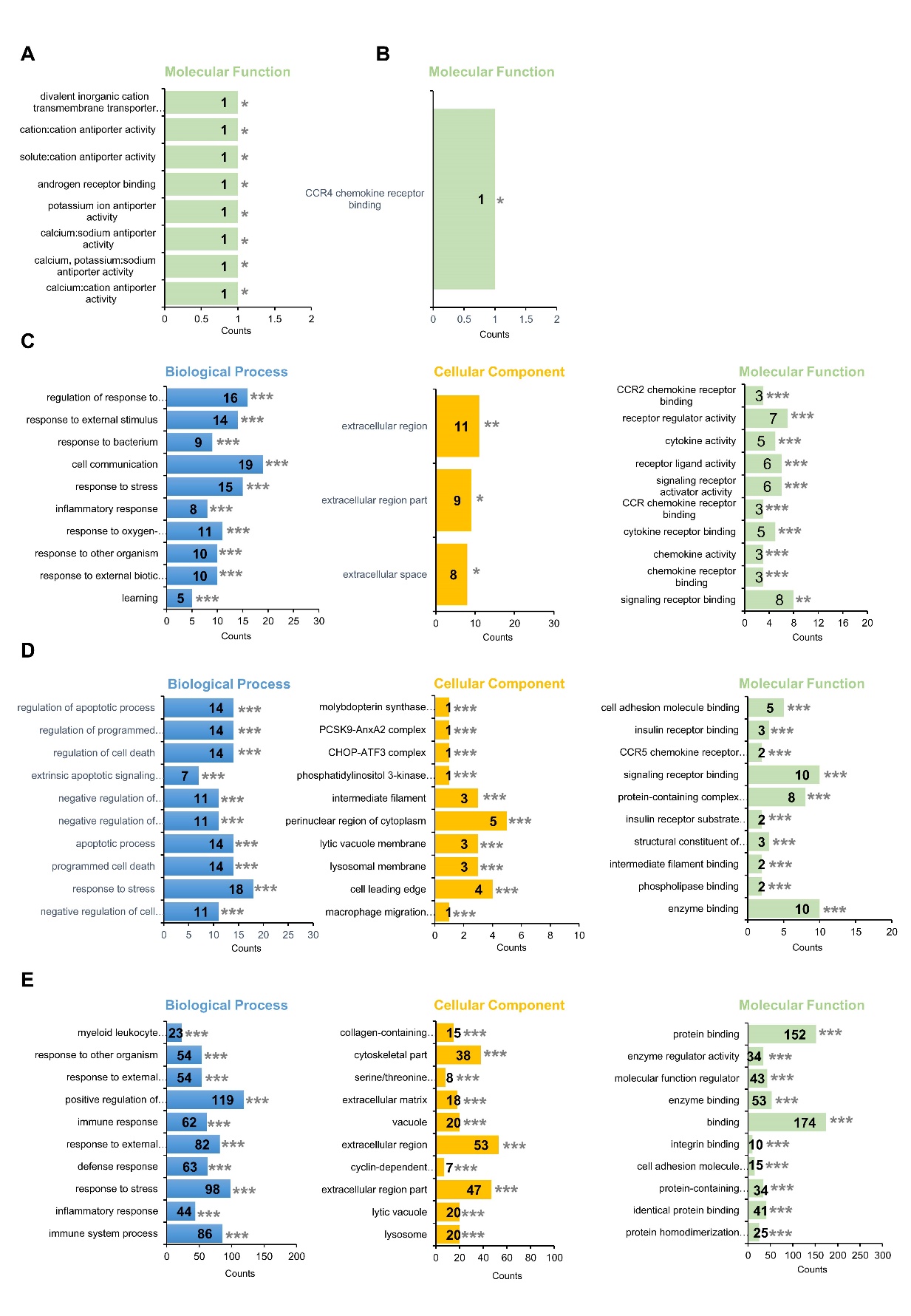


**Additional file 1: Figure S5. Functional enrichment analysis of highly regulated differentially expressed genes (DEGs) in 0.42 MPa condition**

GO functional analysis of DEGs at 1 h (**a**), 6 h (**b**), 12 h (**c**), 24 h (**d**), and 48 h (**e**) compared with sham control. They were annotated using three ontology categories: biological process (blue), cellular component (yellow), and molecular function (green). (**p* < 0.05, ***p* < 0.01 and, ****p* < 0.001).


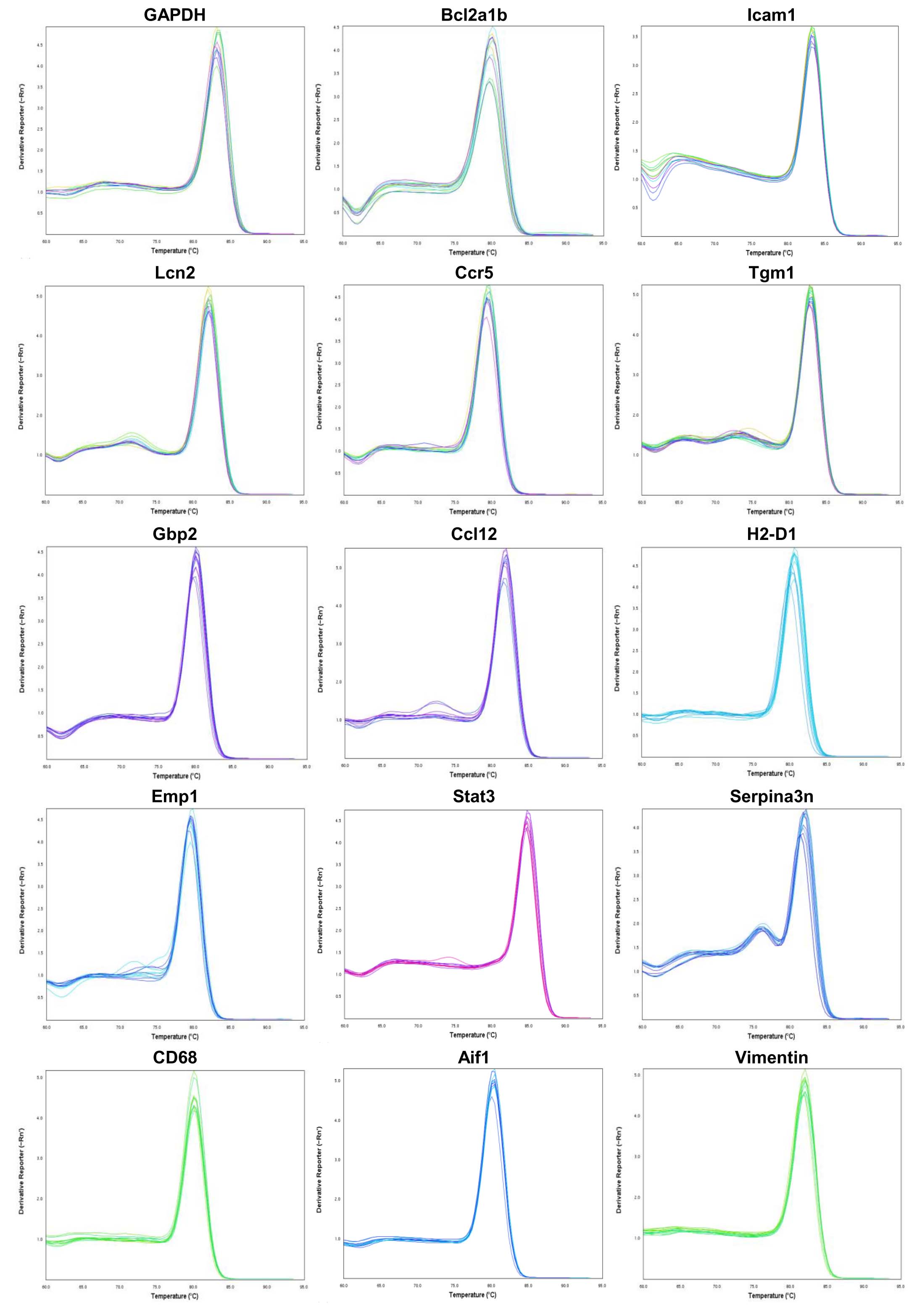


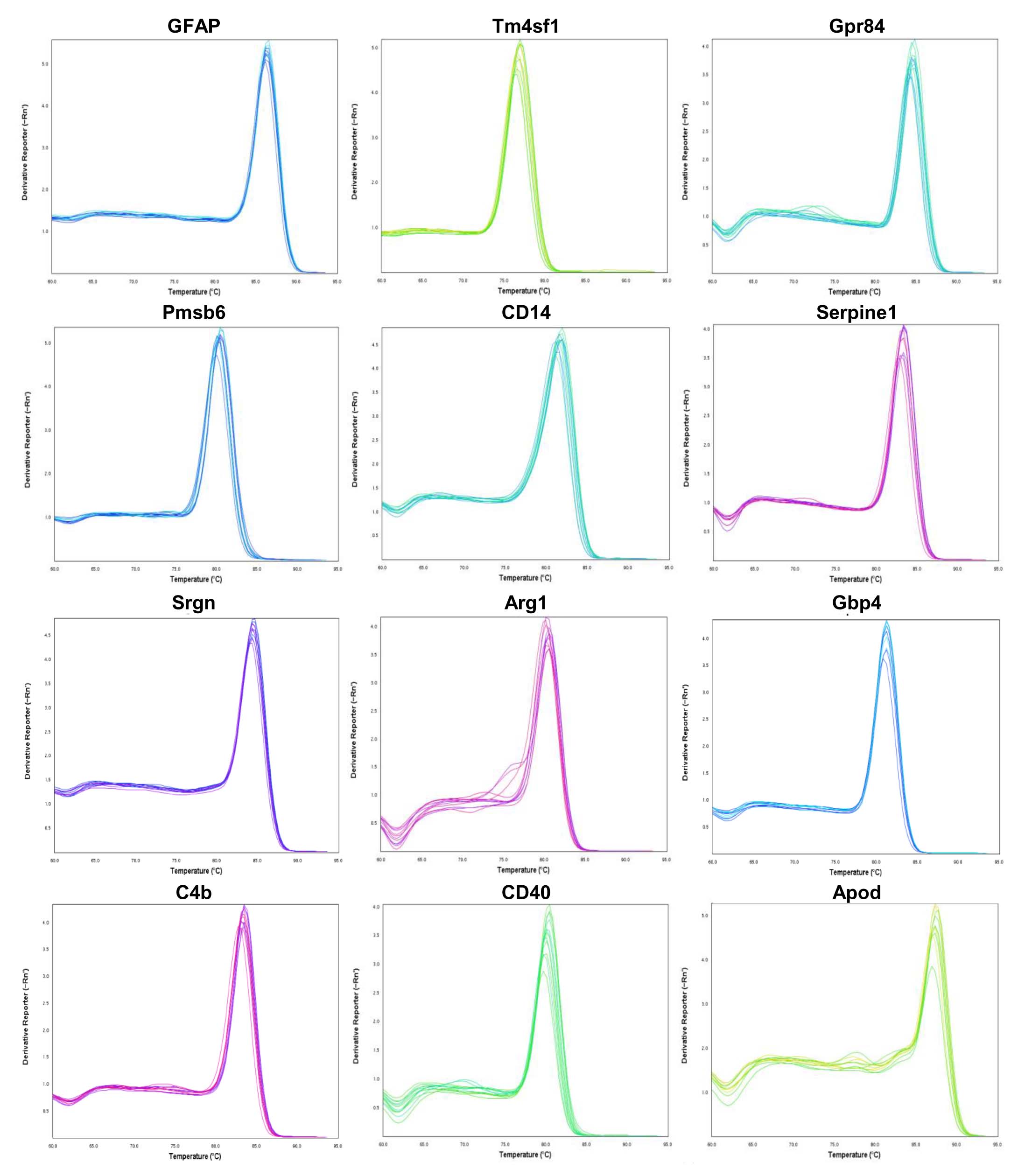


**Figure S6. Melting curve analysis with specificity of RT-qPCR amplification**


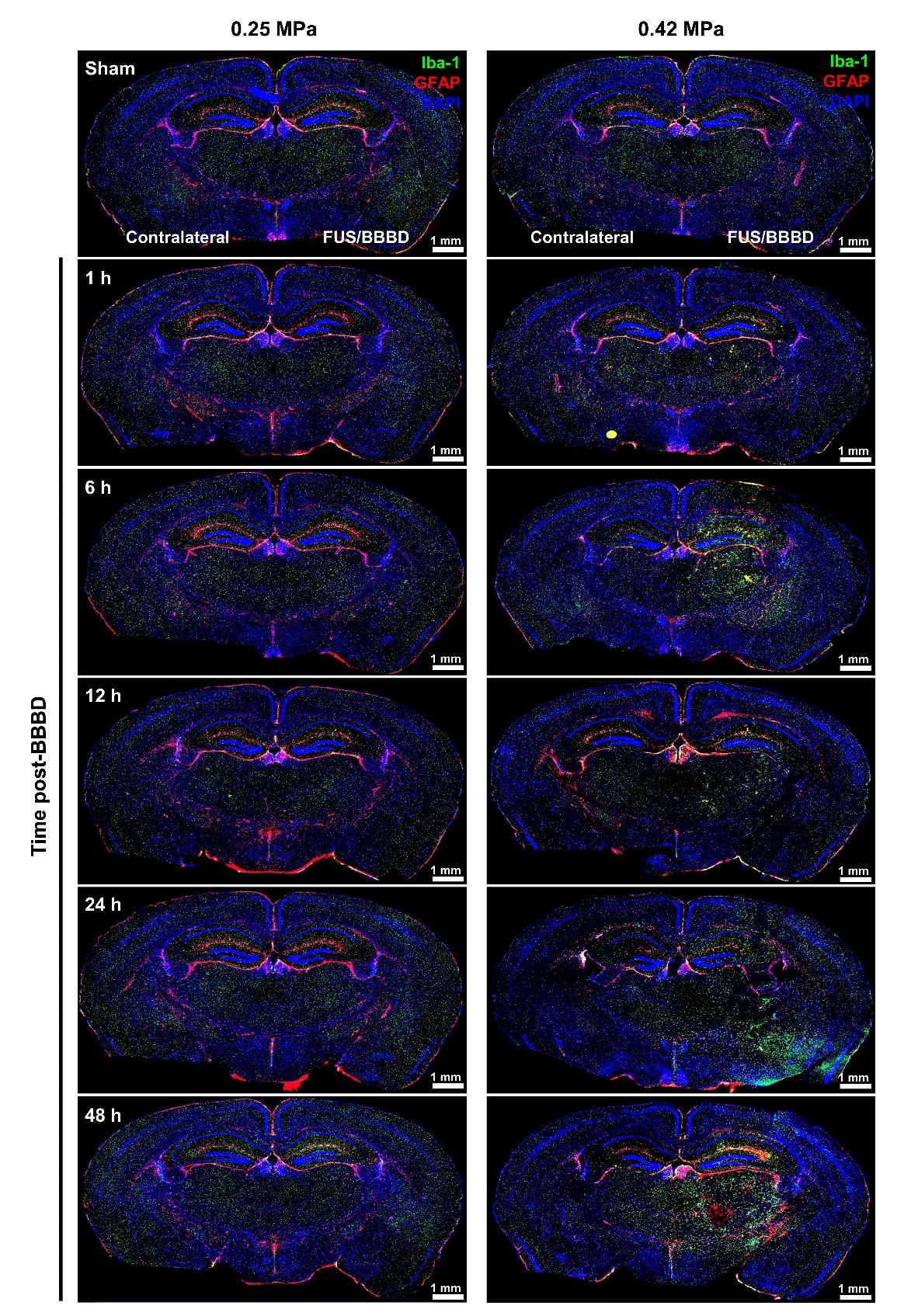


**Additional file 1: Figure S7. The represented whole-brain sections for IF analysis of Iba-1 and GFAP post-BBBD**

Representative brain section images were stained for Iba-1 (green), GFAP (red), and DAPI (blue) at different time points (1, 6, 12, 24, and 48 h). The left and right panels represent the entire brain section for the 0.25 MPa and 0.42 MPa FUS parameter-mediated BBBD, respectively. An increase in green fluorescence (IBA-1-positive microglial cells) was observed in the FUS-treated hemisphere from 6 h to 48 h (right panel, 0.42 MPa). The scale bar represents 1 mm.

**Additional file 1:** Table S1. The differential expressed genes in the 0.25 MPa condition

|  |  | 1 hr | | 6 hr | | 12 hr | | 24 hr | | 48 hr | |
| --- | --- | --- | --- | --- | --- | --- | --- | --- | --- | --- | --- |
| Gene ID | Gene symbol | Log2 FC | P-value | Log2 FC | P-value | Log2 FC | P-value | Log2 FC | P-value | Log2 FC | P-value |
| | 1228 | | --- | | 12575 | | 12608 | | 12785 | | 13653 | | 14281 | | 15130 | | 16007 | | 18035 | | 22695 | | 22785 | | 57435 | | 58801 | | 66511 | | 69623 | | 71878 | | 74132 | | 102032 | | 103466 | | 109801 | | 330460 | | 384864 | | 665931 | | 102466257 | | | Cacb4 | | --- | | Cdkn1a | | Cebpb | | Cnbp | | Egr1 | | Fos | | Hbb-b2 | | Cyr61 | | Nfkbia | | Zfp36 | | Slc30a4 | | Plin4 | | Pmaip1 | | Chtop | | Zfp33b | | Fam83d | | Rnf6 | | Smim19 | | Nt5dc3 | | Glo1 | | Tmem150b | | Gm1943 | | Rplp2-ps1 | | Mir8114 | | | 1.05 | | --- | | -1.09 | | -1.15 | | -1.82 | | -1.04 | | 1.06 | | **-5.24** | | -1.10 | | -1.18 | | 1.17 | | 1.02 | | 1.73 | | -1.84 | | 1.43 | | 1.74 | | -1.16 | | -1.33 | | -1.11 | | 1.09 | | -1.00 | | **2.05** | | 1.15 | | **-2.24** | | **-2.40** | | | 0.55 | | --- | | 0.80 | | 0.37 | | 0.44 | | 0.80 | | 0.81 | | 0.03 | | 0.64 | | 0.06 | | 0.18 | | 0.83 | | 0.28 | | 0.38 | | 0.19 | | 0.49 | | 0.09 | | 0.49 | | 0.19 | | 0.19 | | 0.99 | | 0.04 | | 0.45 | | 0.04 | | 0.02 | | | **-2.58** | | --- | | **2.45** | | **-2.17** | | **-2.34** | | **-2.30** | | -1.12 | | **-3.74** | | -1.64 | | -1.03 | | -1.20 | | -1.01 | | 1.59 | | -1.10 | | 1.13 | | **2.32** | | -1.05 | | **-2.04** | | -1.01 | | 1.95 | | 1.27 | | **2.05** | | **2.51** | | -1.43 | | **3.00** | | | 0.03 | | --- | | 0.03 | | 0.04 | | 0.00 | | 0.02 | | 0.67 | | 0.09 | | 0.41 | | 0.77 | | 0.37 | | 0.96 | | 0.12 | | 0.39 | | 0.17 | | 0.01 | | 0.28 | | 0.03 | | 0.88 | | 0.08 | | 0.27 | | 0.05 | | 0.03 | | 0.50 | | 0.02 | | | -1.05 | | --- | | 1.70 | | -1.10 | | -1.01 | | -1.46 | | -1.65 | | **-3.12** | | **-3.09** | | -1.90 | | -1.73 | | **-2.66** | | **3.86** | | -1.29 | | 1.52 | | 1.02 | | -1.09 | | 1.27 | | **-2.55** | | **2.01** | | **-3.02** | | 1.19 | | 1.03 | | -1.90 | | **2.50** | | | 0.54 | | --- | | 0.57 | | 0.63 | | 0.87 | | 0.23 | | 0.41 | | 0.42 | | 0.08 | | 0.18 | | 0.05 | | 0.01 | | 0.02 | | 0.53 | | 0.26 | | 0.55 | | 0.43 | | 0.22 | | 0.00 | | 0.01 | | 0.03 | | 0.54 | | 0.85 | | 0.09 | | 0.11 | | | 1.02 | | --- | | -1.96 | | -1.20 | | **-2.92** | | **-2.17** | | **-2.94** | | **-6.48** | | **-3.08** | | **-2.14** | | **-2.19** | | 1.02 | | -1.13 | | **-2.14** | | 1.18 | | 1.63 | | **-2.29** | | -1.32 | | -1.02 | | 1.79 | | -1.15 | | 1.65 | | 1.52 | | **-2.01** | | **2.00** | | | 0.77 | | --- | | 0.07 | | 0.40 | | 0.00 | | 0.02 | | 0.02 | | 0.08 | | 0.00 | | 0.01 | | 0.05 | | 0.72 | | 0.62 | | 0.01 | | 0.14 | | 0.47 | | 0.02 | | 0.46 | | 0.78 | | 0.01 | | 0.43 | | 0.19 | | 0.37 | | 0.06 | | 0.26 | | | -1.00 | | --- | | **-2.70** | | -1.29 | | -1.72 | | **-2.22** | | **-2.55** | | **-2.44** | | **-3.50** | | **-2.60** | | **-2.07** | | -1.58 | | -1.59 | | **-2.80** | | **2.19** | | 1.05 | | **-2.31** | | 1.12 | | -1.48 | | -1.07 | | 1.30 | | 1.79 | | 1.12 | | -1.53 | | -1.01 | | | 0.98 | | --- | | 0.03 | | 0.43 | | 0.45 | | 0.23 | | 0.06 | | 0.39 | | 0.01 | | 0.03 | | 0.04 | | 0.51 | | 0.07 | | 0.16 | | 0.01 | | 0.19 | | 0.00 | | 0.49 | | 0.37 | | 0.08 | | 0.24 | | 0.07 | | 0.52 | | 0.53 | | 0.97 | |

**Additional file 1: Table S2**. The differential expressed genes in the 0.42 MPa condition

|  |  | 1 hr | | 6 hr | | 12 hr | | 24 hr | | 48 hr | |
| --- | --- | --- | --- | --- | --- | --- | --- | --- | --- | --- | --- |
| Gene ID | Gene symbol | Log2 FC | P-value | Log2 FC | P-value | Log2 FC | P-value | Log2 FC | P-value | Log2 FC | P-value |
| | 11501 | | --- | | 11520 | | 11690 | | 11745 | | 11799 | | 11810 | | 11838 | | 11853 | | 11857 | | 11867 | | 11910 | | 12044 | | 12045 | | 12047 | | 12051 | | 12064 | | 12229 | | 12236 | | 12257 | | 12259 | | 12260 | | 12262 | | 12267 | | 12268 | | 12273 | | 12306 | | 12332 | | 12428 | | 12442 | | 12443 | | 12505 | | 12506 | | 12508 | | 12512 | | 12514 | | 12523 | | 12534 | | 12545 | | 12575 | | 12642 | | 12654 | | 12702 | | 12774 | | 12798 | | 12826 | | 12983 | | 13011 | | 13024 | | 13032 | | 13057 | | 13610 | | 13685 | | 13732 | | 13733 | | 14127 | | 14129 | | 14325 | | 14580 | | 14594 | | 14619 | | 14663 | | 14727 | | 14728 | | 14793 | | 14824 | | 15109 | | 15162 | | 15163 | | 15170 | | 15368 | | 15894 | | 15900 | | 15939 | | 15944 | | 16391 | | 16414 | | 16792 | | 16819 | | 16852 | | 16854 | | 16859 | | 16913 | | 17064 | | 17079 | | 17084 | | 17096 | | 17105 | | 17112 | | 17215 | | 17216 | | 17218 | | 17219 | | 17345 | | 17392 | | 17434 | | 17476 | | 17698 | | 17916 | | 17969 | | 17972 | | 18008 | | 18301 | | 18607 | | 18708 | | 18792 | | 18793 | | 18816 | | 18817 | | 18824 | | 18826 | | 18938 | | 18950 | | 19128 | | 19141 | | 19144 | | 19225 | | 19247 | | 19354 | | 20135 | | 20195 | | 20200 | | 20202 | | 20288 | | 20293 | | 20295 | | 20296 | | 20302 | | 20303 | | 20306 | | 20375 | | 20491 | | 20716 | | 20750 | | 20753 | | 20848 | | 20877 | | 21345 | | 21346 | | 21391 | | 21803 | | 21810 | | 21816 | | 21847 | | 21857 | | 21937 | | 21946 | | 21973 | | 22177 | | 22271 | | 22324 | | 22352 | | 23833 | | 23880 | | 23882 | | 24110 | | 26388 | | 27279 | | 30794 | | 52033 | | 52276 | | 53324 | | 54124 | | 54519 | | 55932 | | 56089 | | 56193 | | 56743 | | 58223 | | 59126 | | 64138 | | 65221 | | 65972 | | 66058 | | 66141 | | 66197 | | 66442 | | 66589 | | 66824 | | 66929 | | 67052 | | 67844 | | 67896 | | 67951 | | 68026 | | 68545 | | 68612 | | 68662 | | 68774 | | 68800 | | 69453 | | 69550 | | 69774 | | 70466 | | 71994 | | 72119 | | 72318 | | 73656 | | 73804 | | 74091 | | 74132 | | 74178 | | 76376 | | 76905 | | 80876 | | 80885 | | 80891 | | 80910 | | 83433 | | 93695 | | 99543 | | 104816 | | 105855 | | 107321 | | 107373 | | 107581 | | 107995 | | 108052 | | 108101 | | 110006 | | 110033 | | 110454 | | 111241 | | 114584 | | 116701 | | 117167 | | 192187 | | 195434 | | 212032 | | 213393 | | 215387 | | 216161 | | 217203 | | 217333 | | 232345 | | 233406 | | 233571 | | 239743 | | 240832 | | 268697 | | 272551 | | 279572 | | 326618 | | 330460 | | 380732 | | 387208 | | 399591 | | 433855 | | 504193 | | 546546 | | 628900 | | 654820 | | 666899 | | 668218 | | 751865 | | 100034726 | | 100037283 | | 100039192 | | 100526469 | | | Adam8 | | --- | | Plin2 | | Alox5ap | | Anxa3 | | Birc5 | | Apobec1 | | Arc | | Rhoc | | Arhgdib | | Arpc1b | | Atf3 | | Bcl2a1a | | Bcl2a1b | | Bcl2a1d | | Bcl3 | | Bdnf | | Btk | | Bub1b | | Tspo | | C1qa | | C1qb | | C1qc | | C3ar1 | | C4b | | C5ar1 | | Anxa2 | | Capg | | Ccna2 | | Ccnb2 | | Ccnd1 | | Cd44 | | Cd48 | | Cd53 | | Cd63 | | Cd68 | | Cd84 | | Cdk1 | | Cdc7 | | Cdkn1a | | Ch25h | | Chil1 | | Socs3 | | Ccr5 | | Cnn2 | | Col4a1 | | Csf2rb | | Cst7 | | Ctla2a | | Ctsc | | Cyba | | S1pr3 | | Eif4ebp1 | | Emp3 | | Adgre1 | | Fcer1g | | Fcgr1 | | Ftl1 | | Gfap | | Ggta1 | | Gjb2 | | Glycam1 | | Lilr4b | | Lilrb4a | | Cdca3 | | Grn | | Hal | | Hck | | Hcls1 | | Ptpn6 | | Hmox1 | | Icam1 | | Irf8 | | Ier5 | | Irgm1 | | Irf9 | | Itgb2 | | Laptm5 | | Lcn2 | | Lgals1 | | Lgals3 | | Lgals9 | | Psmb8 | | Cd93 | | Cd180 | | Ly86 | | Lyn | | Lyz2 | | Tm4sf1 | | Mcm3 | | Mcm2 | | Mcm5 | | Mcm6 | | Mki67 | | Mmp3 | | Mocs2 | | Mpeg1 | | Msn | | Myo1f | | Ncf1 | | Ncf4 | | Nes | | Fxyd5 | | Pdpk1 | | Pik3r1 | | Plau | | Plaur | | Serpinf2 | | Plk1 | | Plp2 | | Lcp1 | | Ppp1r14b | | Pnp | | Pros1 | | Lgmn | | Klk6 | | Ptgs2 | | Ptpn11 | | Rac2 | | Rrm2 | | S100a11 | | S100a6 | | S100a9 | | Msr1 | | Ccl12 | | Ccl17 | | Ccl2 | | Ccl3 | | Ccl4 | | Ccl7 | | Spi1 | | Sla | | Serpina3n | | Spp1 | | Sprr1a | | Stat3 | | Aurkb | | Tagln | | Tagln2 | | Tbxas1 | | Tgfb1 | | Tgfbi | | Tgm1 | | Klf10 | | Timp1 | | Tnfrsf1a | | Pglyrp1 | | Top2a | | Tyrobp | | Upp1 | | Vav1 | | Vim | | Cd52 | | Fyb | | Gadd45g | | Usp18 | | Ifi202b | | Tnfrsf12a | | Pdlim4 | | Pbk | | Cdca8 | | Nptx2 | | Cks1b | | Apbb1ip | | Gbp3 | | Ramp3 | | Plek | | Lat2 | | Mmp19 | | Nek6 | | Ctsz | | Slc15a3 | | Ifi30 | | Tmem176a | | Ifitm3 | | Cks2 | | Spc25 | | Ube2v1 | | Pycard | | Asf1b | | Ndc80 | | Rab32 | | Ccdc80 | | Tubb6 | | 2810417H13Rik | | Ecscr | | Ube2c | | Scgb3a1 | | Ms4a6d | | Prr32 | | Prss56 | | Bst2 | | Ms4a6b | | Ckap2l | | Cnn3 | | Tpx2 | | Cyth4 | | Ms4a6c | | Kif2c | | Npl | | Rnf6 | | Stk40 | | Slc24a2 | | Lrg1 | | Ifitm2 | | Hcar2 | | Fcrls | | Gpr84 | | Trem2 | | Gpnmb | | Olfml3 | | Aspg | | Nckap1l | | Lpxn | | Fam111a | | Col16a1 | | Cdc20 | | Slc14a1 | | Fermt3 | | Gusb | | Kif22 | | Ly6a | | Hmga1-rs1 | | Clic1 | | Fgfrl1 | | Steap4 | | Stab1 | | Utp14b | | Hk3 | | 8430408G22Rik | | Ncaph | | Sbno2 | | Tmem106a | | Trim47 | | A2m | | Prc1 | | P2ry6 | | Klhl6 | | Tor1aip2 | | Ccnb1 | | Gins2 | | Tlr13 | | Tpm4 | | Tmem150b | | Milr1 | | Mir212 | | Tmsb15l | | AI506816 | | Npcd | | Serpina3h | | Serpina3i | | G530011O06Rik | | Gm12191 | | Bin2 | | Sap25 | | Gm15772 | | Rnaset2a | | Tmem254c | | Mir3064 | | | -1.07 | | --- | | 1.06 | | 1.14 | | -1.17 | | 1.04 | | -1.01 | | 1.55 | | 1.07 | | -1.41 | | -1.10 | | 1.07 | | 1.01 | | 1.13 | | -1.08 | | 1.04 | | 1.03 | | 1.11 | | -1.05 | | -1.20 | | 1.04 | | -1.04 | | 1.09 | | -1.10 | | 1.13 | | -1.02 | | 1.02 | | 1.00 | | -1.06 | | 1.13 | | -1.06 | | 1.21 | | 1.17 | | -1.08 | | 1.06 | | -1.10 | | -1.04 | | 1.04 | | 1.09 | | 1.01 | | -1.09 | | 1.09 | | 1.16 | | 1.08 | | -1.14 | | 1.02 | | 1.05 | | -1.02 | | 1.06 | | 1.03 | | 1.05 | | -1.06 | | -1.15 | | -1.22 | | 1.02 | | 1.07 | | 1.04 | | -1.00 | | 1.13 | | 1.06 | | 1.17 | | -1.09 | | 1.00 | | -1.05 | | 1.00 | | 1.05 | | -1.01 | | -1.05 | | 1.01 | | -1.10 | | -1.00 | | -1.02 | | -1.01 | | -1.09 | | 1.12 | | 1.21 | | -1.05 | | -1.02 | | -1.18 | | -1.08 | | -1.10 | | 1.09 | | -1.09 | | 1.01 | | -1.19 | | -1.06 | | -1.03 | | 1.12 | | -1.14 | | 1.07 | | 1.01 | | -1.06 | | 1.05 | | -1.05 | | 1.00 | | 1.52 | | -1.03 | | -1.05 | | 1.01 | | -1.10 | | 1.18 | | -1.03 | | 1.09 | | -1.06 | | -1.59 | | 1.10 | | -1.03 | | 1.13 | | -1.03 | | -1.05 | | 1.07 | | -1.02 | | 1.09 | | 1.00 | | 1.06 | | 1.03 | | -1.00 | | -1.02 | | 1.04 | | -1.03 | | 1.03 | | 1.02 | | 1.35 | | 1.01 | | 1.17 | | -1.15 | | 1.15 | | -1.11 | | 1.02 | | 1.20 | | -1.02 | | -1.21 | | 1.13 | | -1.13 | | -1.05 | | 1.08 | | -1.11 | | -1.11 | | -1.06 | | 1.01 | | -1.07 | | -1.13 | | 1.04 | | -1.05 | | 1.07 | | 1.13 | | -1.40 | | 1.08 | | 1.04 | | -1.03 | | 1.02 | | -1.12 | | 1.13 | | -1.05 | | 1.32 | | 1.30 | | 1.09 | | 1.12 | | -1.04 | | 1.05 | | -1.00 | | 1.05 | | 1.15 | | -1.05 | | 1.33 | | 1.50 | | -1.11 | | -1.11 | | 1.06 | | -1.09 | | 1.03 | | -1.04 | | -1.02 | | 1.08 | | 1.11 | | 1.10 | | -1.00 | | -1.78 | | -1.04 | | 1.07 | | 1.09 | | 1.04 | | -1.17 | | -1.11 | | 1.03 | | 1.12 | | 1.12 | | 1.00 | | 1.00 | | -1.53 | | -1.71 | | 1.11 | | -1.14 | | -1.03 | | -1.02 | | 1.02 | | -1.01 | | -1.12 | | 1.04 | | -1.09 | | **-2.41** | | 1.07 | | **3.04** | | 1.01 | | -1.07 | | -1.06 | | -1.41 | | 1.05 | | 1.26 | | 1.10 | | -1.11 | | 1.11 | | 1.01 | | -1.04 | | 1.09 | | 1.07 | | 1.13 | | 1.11 | | 1.03 | | -1.01 | | 1.00 | | 1.10 | | 1.24 | | -1.01 | | 1.04 | | -1.06 | | -1.07 | | 1.37 | | 1.04 | | 1.31 | | -1.05 | | 1.01 | | 1.20 | | -1.05 | | -1.09 | | 1.01 | | 1.10 | | -1.05 | | 1.60 | | 1.00 | | 1.11 | | -1.12 | | 1.05 | | **2.15** | | -1.01 | | -1.14 | | -1.88 | | 1.29 | | 1.19 | | -1.02 | | 1.02 | | 1.06 | | -1.05 | | 1.06 | | **2.28** | | **-3.79** | | 1.36 | | -1.60 | | 1.09 | | | 0.51 | | --- | | 0.54 | | 0.49 | | 0.17 | | 0.45 | | 0.85 | | 0.41 | | 0.16 | | 0.04 | | 0.63 | | 0.42 | | 0.65 | | 0.09 | | 0.42 | | 0.74 | | 0.70 | | 0.31 | | 0.28 | | 0.07 | | 0.70 | | 0.70 | | 0.50 | | 0.05 | | 0.11 | | 0.85 | | 0.83 | | 0.98 | | 0.54 | | 0.26 | | 0.64 | | 0.13 | | 0.13 | | 0.57 | | 0.12 | | 0.22 | | 0.29 | | 0.63 | | 0.29 | | 0.98 | | 0.50 | | 0.52 | | 0.20 | | 0.35 | | 0.43 | | 0.81 | | 0.59 | | 0.79 | | 0.74 | | 0.50 | | 0.55 | | 0.29 | | 0.30 | | 0.23 | | 0.87 | | 0.48 | | 0.83 | | 0.94 | | 0.48 | | 0.49 | | 0.58 | | 0.45 | | 1.00 | | 0.61 | | 0.99 | | 0.53 | | 0.44 | | 0.13 | | 0.95 | | 0.19 | | 1.00 | | 0.72 | | 0.91 | | 0.61 | | 0.27 | | 0.10 | | 0.62 | | 0.61 | | 0.44 | | 0.51 | | 0.57 | | 0.50 | | 0.16 | | 0.76 | | 0.03 | | 0.61 | | 0.64 | | 0.58 | | 0.25 | | 0.35 | | 0.90 | | 0.41 | | 0.33 | | 0.60 | | 1.00 | | 0.15 | | 0.87 | | 0.54 | | 0.82 | | 0.35 | | 0.45 | | 0.78 | | 0.51 | | 0.45 | | 0.45 | | 0.33 | | 0.79 | | 0.26 | | 0.60 | | 0.63 | | 0.37 | | 0.85 | | 0.12 | | 0.99 | | 0.34 | | 0.77 | | 0.99 | | 0.74 | | 0.60 | | 0.67 | | 0.92 | | 0.91 | | 0.41 | | 0.83 | | 0.27 | | 0.54 | | 0.31 | | 0.65 | | 0.89 | | 0.31 | | 0.83 | | 0.20 | | 0.38 | | 0.69 | | 0.48 | | 0.39 | | 0.29 | | 0.71 | | 0.64 | | 0.91 | | 0.59 | | 0.14 | | 0.74 | | 0.69 | | 0.46 | | 0.04 | | 0.20 | | 0.58 | | 0.50 | | 0.73 | | 0.80 | | 0.31 | | 0.53 | | 0.55 | | 0.17 | | 0.41 | | 0.33 | | 0.38 | | 0.60 | | 0.16 | | 1.00 | | 0.80 | | 0.05 | | 0.50 | | 0.14 | | 0.09 | | 0.32 | | 0.18 | | 0.22 | | 0.03 | | 0.70 | | 0.52 | | 0.93 | | 0.37 | | 0.49 | | 0.49 | | 0.96 | | 0.52 | | 0.72 | | 0.09 | | 0.34 | | 0.65 | | 0.02 | | 0.58 | | 0.69 | | 0.42 | | 0.41 | | 0.98 | | 1.00 | | 0.30 | | 0.17 | | 0.46 | | 0.48 | | 0.54 | | 0.74 | | 0.74 | | 0.94 | | 0.07 | | 0.47 | | 0.52 | | 0.02 | | 0.58 | | 0.02 | | 0.95 | | 0.67 | | 0.10 | | 0.48 | | 0.64 | | 0.49 | | 0.21 | | 0.42 | | 0.50 | | 0.92 | | 0.63 | | 0.15 | | 0.58 | | 0.00 | | 0.51 | | 0.81 | | 0.70 | | 0.99 | | 0.09 | | 0.29 | | 0.84 | | 0.58 | | 0.22 | | 0.68 | | 0.14 | | 0.10 | | 0.15 | | 0.38 | | 0.77 | | 0.28 | | 0.62 | | 0.44 | | 0.91 | | 0.34 | | 0.38 | | 0.39 | | 0.99 | | 0.27 | | 0.12 | | 0.44 | | 0.05 | | 0.80 | | 0.37 | | 0.06 | | 0.53 | | 0.55 | | 0.77 | | 0.61 | | 0.74 | | 0.94 | | 0.63 | | 0.21 | | 0.04 | | 0.25 | | 0.35 | | 0.29 | | | -1.01 | | --- | | 1.35 | | -1.07 | | 1.00 | | -1.04 | | 1.53 | | 1.98 | | 1.45 | | -1.57 | | -1.09 | | 1.82 | | 1.06 | | 1.15 | | 1.06 | | 1.44 | | 1.23 | | -1.01 | | -1.10 | | -1.22 | | 1.02 | | 1.03 | | 1.07 | | 1.24 | | 1.09 | | 1.38 | | 1.13 | | 1.23 | | 1.05 | | 1.14 | | -1.08 | | 1.08 | | -1.11 | | -1.05 | | -1.02 | | -1.20 | | -1.14 | | -1.01 | | **-2.07** | | **4.46** | | 1.70 | | 1.09 | | 1.96 | | 1.22 | | 1.10 | | 1.35 | | 1.22 | | 1.03 | | **2.89** | | -1.04 | | -1.02 | | -1.03 | | -1.11 | | -1.05 | | -1.27 | | -1.20 | | 1.01 | | 1.13 | | 1.28 | | 1.34 | | 1.81 | | 1.05 | | 1.10 | | 1.03 | | -1.04 | | -1.06 | | -1.00 | | -1.09 | | -1.12 | | -1.11 | | **2.08** | | 1.74 | | -1.07 | | 1.45 | | 1.52 | | 1.25 | | -1.09 | | -1.18 | | **4.32** | | -1.17 | | 1.07 | | 1.18 | | -1.46 | | 1.50 | | -1.11 | | -1.10 | | 1.02 | | 1.03 | | -1.09 | | 1.09 | | 1.37 | | -1.17 | | -1.01 | | -1.06 | | 1.02 | | 1.30 | | -1.57 | | 1.15 | | -1.13 | | 1.05 | | -1.01 | | 1.17 | | 1.26 | | -1.06 | | -1.70 | | 1.48 | | **2.23** | | 1.12 | | 1.01 | | -1.18 | | -1.32 | | -1.05 | | 1.02 | | -1.05 | | 1.03 | | 1.10 | | **2.19** | | 1.15 | | 1.03 | | 1.35 | | -1.06 | | 1.01 | | 1.22 | | 1.11 | | **2.30** | | **2.22** | | **3.32** | | **3.09** | | **3.68** | | **2.21** | | -1.05 | | -1.05 | | 1.19 | | 1.19 | | -1.07 | | 1.43 | | 1.02 | | 1.17 | | 1.15 | | -1.15 | | -1.02 | | -1.19 | | 1.03 | | 1.39 | | 1.22 | | 1.22 | | 1.58 | | -1.07 | | 1.02 | | 1.14 | | 1.06 | | 1.01 | | -1.01 | | 1.08 | | **3.43** | | 1.02 | | 1.30 | | 1.78 | | -1.06 | | -1.08 | | -1.13 | | 1.52 | | -1.11 | | 1.02 | | 1.04 | | -1.13 | | 1.33 | | -1.17 | | 1.08 | | 1.03 | | -1.00 | | 1.57 | | 1.20 | | -1.04 | | 1.03 | | -1.02 | | 1.14 | | -1.69 | | -1.19 | | 1.17 | | 1.09 | | 1.20 | | -1.07 | | 1.60 | | 1.00 | | -1.17 | | -1.05 | | -1.00 | | 1.02 | | **-2.19** | | **-2.36** | | -1.20 | | -1.04 | | -1.10 | | 1.08 | | -1.08 | | -1.26 | | 1.03 | | 1.05 | | -1.36 | | **-2.23** | | 1.42 | | -1.01 | | 1.81 | | 1.02 | | 1.33 | | **-2.26** | | 1.40 | | -1.12 | | 1.07 | | -1.52 | | 1.35 | | -1.12 | | 1.08 | | 1.10 | | 1.13 | | 1.26 | | -1.00 | | -1.05 | | -1.12 | | 1.09 | | 1.30 | | 1.56 | | 1.01 | | -1.20 | | 1.05 | | -1.03 | | **2.13** | | -1.26 | | **2.58** | | 1.28 | | 1.46 | | 1.11 | | -1.05 | | 1.10 | | 1.10 | | 1.02 | | 1.04 | | 1.53 | | 1.11 | | 1.05 | | -1.04 | | 1.35 | | 1.76 | | 1.02 | | 1.72 | | **-2.03** | | -1.51 | | **2.35** | | 1.32 | | 1.17 | | 1.37 | | -1.74 | | -1.49 | | 1.01 | | 1.36 | | 1.26 | | -1.34 | | 1.84 | | | 0.95 | | --- | | 0.23 | | 0.67 | | 0.99 | | 0.80 | | 0.10 | | 0.36 | | 0.16 | | 0.10 | | 0.43 | | 0.40 | | 0.40 | | 0.24 | | 0.50 | | 0.12 | | 0.42 | | 0.91 | | 0.18 | | 0.08 | | 0.68 | | 0.85 | | 0.50 | | 0.52 | | 0.53 | | 0.41 | | 0.45 | | 0.25 | | 0.62 | | 0.25 | | 0.35 | | 0.45 | | 0.27 | | 0.68 | | 0.74 | | 0.41 | | 0.24 | | 0.87 | | 0.01 | | 0.11 | | 0.10 | | 0.61 | | 0.29 | | 0.44 | | 0.59 | | 0.19 | | 0.28 | | 0.82 | | 0.27 | | 0.64 | | 0.16 | | 0.61 | | 0.54 | | 0.66 | | 0.33 | | 0.23 | | 0.94 | | 0.53 | | 0.23 | | 0.25 | | 0.31 | | 0.55 | | 0.45 | | 0.42 | | 0.81 | | 0.47 | | 0.89 | | 0.10 | | 0.43 | | 0.45 | | 0.17 | | 0.23 | | 0.41 | | 0.30 | | 0.10 | | 0.14 | | 0.14 | | 0.18 | | 0.27 | | 0.27 | | 0.67 | | 0.27 | | 0.01 | | 0.32 | | 0.56 | | 0.47 | | 0.76 | | 0.92 | | 0.63 | | 0.21 | | 0.05 | | 0.11 | | 0.82 | | 0.55 | | 0.63 | | 0.58 | | 0.16 | | 0.45 | | 0.05 | | 0.53 | | 0.97 | | 0.36 | | 0.25 | | 0.50 | | 0.00 | | 0.06 | | 0.29 | | 0.27 | | 0.91 | | 0.24 | | 0.06 | | 0.57 | | 0.65 | | 0.46 | | 0.48 | | 0.36 | | 0.34 | | 0.27 | | 0.77 | | 0.15 | | 0.82 | | 0.96 | | 0.24 | | 0.43 | | 0.38 | | 0.03 | | 0.17 | | 0.32 | | 0.13 | | 0.25 | | 0.67 | | 0.72 | | 0.25 | | 0.61 | | 0.37 | | 0.08 | | 0.74 | | 0.59 | | 0.46 | | 0.38 | | 0.87 | | 0.45 | | 0.77 | | 0.02 | | 0.11 | | 0.35 | | 0.17 | | 0.63 | | 0.84 | | 0.22 | | 0.49 | | 0.91 | | 0.98 | | 0.46 | | 0.08 | | 0.84 | | 0.12 | | 0.04 | | 0.32 | | 0.14 | | 0.42 | | 0.22 | | 0.39 | | 0.70 | | 0.78 | | 0.56 | | 0.33 | | 0.42 | | 0.54 | | 0.68 | | 0.98 | | 0.21 | | 0.33 | | 0.52 | | 0.82 | | 0.86 | | 0.35 | | 0.46 | | 0.17 | | 0.03 | | 0.23 | | 0.21 | | 0.32 | | 0.24 | | 0.95 | | 0.16 | | 0.70 | | 1.00 | | 0.89 | | 0.04 | | 0.02 | | 0.38 | | 0.70 | | 0.19 | | 0.36 | | 0.30 | | 0.04 | | 0.85 | | 0.35 | | **0.00** | | **0.03** | | 0.24 | | 0.91 | | 0.29 | | 0.94 | | 0.42 | | **0.05** | | 0.45 | | 0.49 | | 0.52 | | 0.15 | | 0.15 | | 0.50 | | 0.31 | | 0.11 | | 0.36 | | 0.10 | | 0.97 | | 0.71 | | **0.02** | | 0.51 | | 0.58 | | 0.16 | | 0.95 | | 0.10 | | 0.80 | | 0.89 | | **0.02** | | **0.03** | | **0.02** | | **0.04** | | 0.23 | | 0.15 | | 0.60 | | 0.52 | | 0.38 | | 0.78 | | 0.83 | | 0.48 | | 0.17 | | 0.53 | | 0.51 | | 0.22 | | 0.30 | | 0.59 | | 0.42 | | 0.00 | | 0.52 | | 0.04 | | 0.03 | | 0.08 | | 0.04 | | 0.48 | | 0.07 | | 0.92 | | 0.43 | | 0.44 | | 0.50 | | 0.31 | | | 1.51 | | --- | | 1.33 | | 1.08 | | 1.21 | | 1.04 | | 1.41 | | **3.63** | | 1.47 | | -1.26 | | 1.34 | | **2.47** | | 1.37 | | 1.52 | | 1.17 | | **2.06** | | **2.10** | | 1.15 | | -1.03 | | 1.15 | | 1.36 | | 1.45 | | 1.47 | | 1.51 | | 1.56 | | 1.72 | | 1.62 | | 1.16 | | -1.02 | | -1.02 | | 1.48 | | 1.68 | | 1.04 | | 1.27 | | 1.18 | | 1.36 | | 1.01 | | -1.05 | | -1.44 | | **3.10** | | **2.36** | | -1.07 | | **2.70** | | **2.02** | | 1.49 | | 1.46 | | 1.57 | | 1.13 | | **4.66** | | 1.23 | | 1.33 | | 1.34 | | 1.21 | | 1.16 | | -1.18 | | 1.20 | | 1.44 | | 1.34 | | 1.89 | | 1.69 | | **2.23** | | 1.93 | | 1.43 | | 1.96 | | -1.12 | | -1.07 | | 1.03 | | 1.31 | | 1.08 | | 1.10 | | **2.75** | | **2.64** | | 1.26 | | **2.15** | | 1.94 | | 1.74 | | 1.08 | | -1.17 | | **11.40** | | -1.00 | | 1.42 | | 1.25 | | 1.30 | | 1.93 | | -1.05 | | 1.06 | | 1.20 | | 1.14 | | 1.13 | | -1.03 | | 1.14 | | -1.19 | | -1.24 | | -1.06 | | 1.36 | | 1.70 | | -1.38 | | 1.48 | | 1.01 | | -1.02 | | 1.80 | | 1.22 | | 1.83 | | 1.01 | | 1.01 | | 1.61 | | **2.56** | | **2.12** | | 1.02 | | 1.02 | | 1.38 | | 1.02 | | 1.32 | | 1.29 | | 1.14 | | -1.31 | | **3.63** | | 1.10 | | 1.32 | | 1.33 | | 1.27 | | 1.25 | | 1.74 | | 1.96 | | **8.71** | | 1.84 | | **6.15** | | **3.80** | | **4.26** | | **2.75** | | 1.33 | | -1.02 | | 1.80 | | 1.66 | | -1.03 | | 1.84 | | -1.10 | | -1.05 | | 1.41 | | -1.14 | | 1.19 | | 1.09 | | 1.55 | | **2.18** | | **2.33** | | **2.03** | | **2.33** | | -1.09 | | 1.15 | | 1.20 | | 1.15 | | 1.39 | | 1.51 | | 1.22 | | **3.12** | | 1.57 | | 1.55 | | **2.17** | | 1.30 | | -1.05 | | -1.09 | | **3.23** | | -1.07 | | 1.28 | | 1.81 | | -1.11 | | 1.35 | | 1.06 | | 1.20 | | 1.19 | | 1.17 | | **2.01** | | 1.32 | | 1.12 | | **2.57** | | -1.13 | | 1.07 | | -1.00 | | 1.24 | | 1.17 | | 1.02 | | 1.34 | | -1.03 | | **2.14** | | -1.01 | | 1.21 | | 1.07 | | 1.26 | | **2.17** | | -1.29 | | -1.24 | | 1.78 | | 1.02 | | -1.12 | | 1.22 | | -1.01 | | 1.19 | | 1.38 | | 1.13 | | -1.04 | | 1.16 | | 1.95 | | **2.72** | | **2.03** | | 1.24 | | 1.94 | | 1.02 | | **2.29** | | -1.30 | | 1.13 | | -1.42 | | 1.98 | | 1.13 | | 1.06 | | 1.34 | | 1.03 | | 1.25 | | 1.46 | | 1.15 | | -1.03 | | 1.01 | | **2.55** | | 1.40 | | 1.31 | | 1.03 | | 1.59 | | -1.11 | | 1.71 | | 1.10 | | 1.78 | | 1.06 | | **2.17** | | 1.39 | | 1.42 | | 1.23 | | -1.09 | | 1.37 | | 1.07 | | 1.49 | | -1.07 | | 1.07 | | 1.00 | | 1.38 | | -1.06 | | 1.18 | | **2.08** | | **-2.74** | | 1.29 | | 1.86 | | 1.77 | | 1.23 | | 2.00 | | -1.31 | | 1.07 | | 1.20 | | **-4.14** | | 1.28 | | -1.73 | | 1.74 | | | 0.32 | | --- | | 0.17 | | 0.70 | | 0.15 | | 0.66 | | 0.09 | | 0.02 | | 0.19 | | 0.35 | | 0.10 | | 0.28 | | 0.30 | | 0.09 | | 0.14 | | 0.11 | | 0.04 | | 0.21 | | 0.45 | | 0.48 | | 0.19 | | 0.24 | | 0.18 | | 0.27 | | 0.14 | | 0.14 | | 0.03 | | 0.32 | | 0.85 | | 0.89 | | 0.01 | | 0.02 | | 0.59 | | 0.14 | | 0.30 | | 0.22 | | 0.91 | | 0.61 | | 0.19 | | 0.04 | | 0.09 | | 0.51 | | 0.17 | | 0.03 | | 0.38 | | 0.01 | | 0.03 | | 0.33 | | 0.02 | | 0.04 | | 0.02 | | 0.35 | | 0.25 | | 0.31 | | 0.06 | | 0.58 | | 0.27 | | 0.02 | | 0.13 | | 0.12 | | 0.04 | | 0.09 | | 0.13 | | 0.00 | | 0.49 | | 0.46 | | 0.18 | | 0.16 | | 0.68 | | 0.59 | | 0.09 | | 0.09 | | 0.24 | | 0.01 | | 0.04 | | 0.17 | | 0.28 | | 0.08 | | 0.02 | | 0.99 | | 0.18 | | 0.54 | | 0.58 | | 0.01 | | 0.67 | | 0.72 | | 0.40 | | 0.58 | | 0.59 | | 0.79 | | 0.44 | | 0.10 | | 0.13 | | 0.57 | | 0.07 | | 0.02 | | 0.50 | | 0.06 | | 0.85 | | 0.80 | | 0.10 | | 0.27 | | 0.10 | | 0.89 | | 0.89 | | 0.03 | | 0.12 | | 0.05 | | 0.74 | | 0.87 | | 0.09 | | 0.81 | | 0.14 | | 0.05 | | 0.11 | | 0.27 | | 0.00 | | 0.26 | | 0.02 | | 0.17 | | 0.43 | | 0.38 | | 0.01 | | 0.04 | | 0.00 | | 0.08 | | 0.02 | | 0.12 | | 0.09 | | 0.04 | | 0.17 | | 0.91 | | 0.08 | | 0.26 | | 0.62 | | 0.02 | | 0.33 | | 0.89 | | 0.22 | | 0.37 | | 0.32 | | 0.71 | | 0.22 | | 0.03 | | 0.01 | | 0.06 | | 0.04 | | 0.54 | | 0.33 | | 0.29 | | 0.18 | | 0.22 | | 0.15 | | 0.12 | | 0.03 | | 0.49 | | 0.19 | | 0.05 | | 0.03 | | 0.72 | | 0.54 | | 0.03 | | 0.30 | | 0.07 | | 0.27 | | 0.12 | | 0.28 | | 0.70 | | 0.04 | | 0.01 | | 0.15 | | 0.08 | | 0.21 | | 0.43 | | 0.22 | | 0.41 | | 0.34 | | 0.95 | | 0.32 | | 0.10 | | 0.64 | | 0.08 | | 0.14 | | 0.09 | | 0.89 | | 0.11 | | 0.61 | | 0.20 | | 0.28 | | 0.42 | | 0.53 | | 0.47 | | 0.94 | | 0.15 | | 0.17 | | 0.83 | | 0.05 | | 0.49 | | 0.25 | | 0.24 | | 0.13 | | 0.02 | | 0.04 | | 0.00 | | 0.30 | | 0.25 | | 0.87 | | 0.05 | | 0.20 | | 0.31 | | 0.02 | | 0.04 | | 0.34 | | 0.58 | | 0.15 | | 0.83 | | 0.08 | | 0.15 | | 0.55 | | 0.21 | | 0.64 | | 0.04 | | 0.21 | | 0.15 | | 0.71 | | 0.04 | | 0.55 | | 0.07 | | 0.20 | | 0.10 | | 0.31 | | 0.14 | | 0.16 | | 0.13 | | 0.11 | | 0.45 | | 0.12 | | 0.25 | | 0.53 | | 0.19 | | 0.48 | | 0.93 | | 0.16 | | 0.73 | | 0.24 | | 0.04 | | 0.10 | | 0.37 | | 0.15 | | 0.01 | | 0.04 | | 0.00 | | 0.73 | | 0.55 | | 0.54 | | 0.07 | | 0.19 | | 0.42 | | 0.03 | | | 1.74 | | --- | | 1.81 | | 1.30 | | 1.76 | | -1.10 | | 1.53 | | 2.02 | | 1.69 | | 1.18 | | 1.71 | | **3.16** | | 1.51 | | **2.24** | | 1.77 | | **3.23** | | 1.42 | | 1.23 | | -1.03 | | 1.40 | | 1.57 | | 1.72 | | 1.75 | | 1.69 | | **2.55** | | 1.63 | | **2.01** | | **2.02** | | -1.00 | | 1.14 | | 1.54 | | **2.59** | | 1.43 | | 1.27 | | 1.49 | | 1.79 | | 1.11 | | 1.05 | | -1.36 | | **2.83** | | **3.61** | | 1.81 | | **3.64** | | 1.84 | | 1.78 | | 1.92 | | 1.64 | | 1.41 | | **3.61** | | 1.57 | | 1.62 | | **2.23** | | 1.36 | | 1.75 | | -1.07 | | 1.62 | | 1.58 | | 1.40 | | **3.60** | | 1.94 | | 1.71 | | **2.39** | | 1.55 | | **2.27** | | -1.11 | | 1.04 | | 1.01 | | 1.21 | | 1.22 | | 1.36 | | **6.75** | | **3.16** | | 1.34 | | 1.27 | | **2.25** | | **2.25** | | 1.29 | | 1.03 | | **14.33** | | 1.62 | | 1.96 | | 1.32 | | 1.32 | | 1.87 | | 1.04 | | 1.42 | | 1.30 | | 1.55 | | 1.43 | | 1.38 | | 1.35 | | 1.09 | | -1.01 | | -1.05 | | **2.27** | | **2.03** | | -1.04 | | **2.34** | | 1.02 | | 1.17 | | 1.85 | | **2.10** | | **2.14** | | **-2.98** | | **-2.01** | | 1.50 | | **2.57** | | **3.48** | | 1.03 | | -1.01 | | 1.63 | | 1.36 | | 1.51 | | 1.78 | | 1.13 | | 1.13 | | 1.77 | | **-2.18** | | 1.32 | | 1.03 | | **2.08** | | 1.93 | | 1.48 | | **3.99** | | **10.94** | | -1.07 | | **6.80** | | **3.30** | | **4.41** | | **3.21** | | 1.59 | | 1.25 | | **2.89** | | **4.62** | | 1.19 | | **2.33** | | 1.04 | | 1.77 | | **2.06** | | 1.04 | | 1.34 | | 1.17 | | **7.02** | | 1.68 | | **5.04** | | **2.97** | | 1.60 | | 1.04 | | 1.39 | | 1.51 | | 1.21 | | **2.46** | | 1.90 | | 1.15 | | **2.75** | | **2.10** | | **4.80** | | **2.95** | | 1.89 | | 1.02 | | -1.13 | | 1.15 | | 1.22 | | 1.55 | | 1.89 | | -1.45 | | 1.41 | | 1.21 | | 1.41 | | 1.48 | | 1.40 | | **2.14** | | 1.66 | | 1.42 | | **2.58** | | -1.11 | | 1.19 | | -1.76 | | 1.58 | | 1.13 | | -1.00 | | 1.46 | | 1.38 | | **3.02** | | 1.04 | | 1.44 | | 1.05 | | **2.11** | | **2.62** | | **-2.10** | | **-2.38** | | 1.42 | | 1.51 | | -1.06 | | 1.61 | | -1.08 | | 1.24 | | 1.95 | | 1.16 | | -1.04 | | 1.06 | | **2.01** | | 1.63 | | **2.69** | | 1.54 | | 1.86 | | 1.40 | | 1.97 | | -1.26 | | 1.78 | | -1.21 | | **2.90** | | 1.19 | | 1.33 | | 1.80 | | 1.72 | | 1.06 | | **2.06** | | 1.42 | | 1.13 | | -1.01 | | **2.31** | | 1.50 | | 1.79 | | 1.73 | | **2.53** | | 1.17 | | 1.18 | | 1.29 | | -1.07 | | 1.02 | | **3.21** | | 1.38 | | 1.86 | | **2.01** | | 1.01 | | 1.42 | | 1.31 | | 1.55 | | -1.12 | | 1.40 | | 1.12 | | 1.60 | | 1.81 | | 1.52 | | -1.10 | | -1.75 | | **2.04** | | 1.23 | | **2.95** | | 1.74 | | 1.84 | | -1.97 | | 1.21 | | -1.03 | | 1.05 | | 1.32 | | -1.05 | | **2.09** | | | 0.17 | | --- | | 0.09 | | 0.23 | | 0.04 | | 0.17 | | 0.21 | | 0.27 | | 0.11 | | 0.08 | | 0.07 | | 0.02 | | 0.29 | | 0.03 | | 0.03 | | 0.09 | | 0.17 | | 0.14 | | 0.76 | | 0.09 | | 0.22 | | 0.18 | | 0.20 | | 0.01 | | 0.14 | | 0.36 | | 0.03 | | 0.09 | | 1.00 | | 0.34 | | 0.03 | | 0.02 | | 0.06 | | 0.36 | | 0.12 | | 0.21 | | 0.37 | | 0.63 | | 0.01 | | 0.34 | | 0.10 | | 0.04 | | 0.10 | | 0.10 | | 0.12 | | 0.22 | | 0.14 | | 0.11 | | 0.18 | | 0.02 | | 0.09 | | 0.07 | | 0.34 | | 0.04 | | 0.73 | | 0.15 | | 0.17 | | 0.03 | | 0.02 | | 0.08 | | 0.01 | | 0.02 | | 0.15 | | 0.12 | | 0.56 | | 0.68 | | 0.48 | | 0.18 | | 0.19 | | 0.04 | | 0.02 | | 0.00 | | 0.12 | | 0.28 | | 0.17 | | 0.19 | | 0.10 | | 0.72 | | 0.23 | | 0.10 | | 0.07 | | 0.36 | | 0.37 | | 0.13 | | 0.73 | | 0.15 | | 0.10 | | 0.18 | | **0.04** | | 0.31 | | 0.23 | | 0.73 | | 0.82 | | 0.66 | | 0.17 | | **0.04** | | 0.84 | | 0.06 | | 0.80 | | 0.42 | | 0.08 | | **0.02** | | 0.14 | | **0.01** | | **0.03** | | **0.04** | | 0.12 | | **0.02** | | 0.60 | | 0.95 | | 0.10 | | 0.08 | | **0.03** | | 0.07 | | 0.10 | | 0.59 | | 0.13 | | **0.05** | | **0.02** | | 0.88 | | 0.12 | | 0.14 | | 0.30 | | 0.09 | | 0.08 | | 0.71 | | 0.20 | | 0.11 | | 0.06 | | 0.23 | | 0.11 | | 0.20 | | 0.24 | | **0.04** | | 0.10 | | **0.02** | | 0.67 | | 0.10 | | **0.03** | | 0.73 | | 0.15 | | 0.52 | | 0.07 | | 0.15 | | **0.02** | | 0.09 | | 0.47 | | 0.88 | | 0.12 | | 0.29 | | 0.14 | | **0.03** | | 0.11 | | 0.19 | | **0.02** | | 0.40 | | 0.09 | | 0.01 | | 0.01 | | 0.93 | | 0.55 | | 0.58 | | 0.16 | | 0.11 | | 0.17 | | 0.48 | | 0.16 | | 0.26 | | 0.21 | | 0.15 | | 0.07 | | 0.04 | | 0.08 | | 0.14 | | 0.14 | | 0.44 | | 0.03 | | 0.50 | | 0.03 | | 0.17 | | 0.91 | | 0.07 | | 0.21 | | 0.04 | | 0.67 | | 0.07 | | 0.74 | | 0.05 | | 0.37 | | 0.11 | | 0.01 | | 0.50 | | 0.50 | | 0.47 | | 0.01 | | 0.60 | | 0.06 | | 0.42 | | 0.14 | | 0.41 | | 0.41 | | 0.01 | | 0.55 | | 0.22 | | 0.31 | | 0.13 | | 0.39 | | 0.09 | | 0.34 | | 0.01 | | 0.59 | | 0.09 | | 0.25 | | 0.07 | | 0.00 | | 0.16 | | 0.57 | | 0.05 | | 0.13 | | 0.24 | | 0.76 | | 0.16 | | 0.17 | | 0.04 | | 0.03 | | 0.09 | | 0.56 | | 0.34 | | 0.05 | | 0.72 | | 0.68 | | 0.02 | | 0.01 | | 0.06 | | 0.18 | | 0.93 | | 0.10 | | 0.13 | | 0.49 | | 0.27 | | 0.21 | | 0.24 | | 0.09 | | 0.06 | | 0.06 | | 0.62 | | 0.26 | | 0.01 | | 0.19 | | 0.24 | | 0.23 | | 0.01 | | 0.30 | | 0.40 | | 0.54 | | 0.57 | | 0.13 | | 0.85 | | 0.21 | | | **3.54** | | --- | | **5.18** | | **2.76** | | **3.31** | | **4.07** | | **4.52** | | 1.54 | | **2.41** | | **3.02** | | **3.65** | | **3.13** | | **2.08** | | **4.31** | | **2.69** | | **3.82** | | -1.14 | | **2.10** | | **2.50** | | **3.25** | | **3.21** | | **3.88** | | **4.54** | | **3.87** | | **5.85** | | **2.06** | | **3.90** | | **5.16** | | **3.20** | | **3.88** | | **2.50** | | **3.24** | | **2.33** | | **2.42** | | **2.54** | | **4.97** | | **2.42** | | **3.46** | | -1.32 | | 1.74 | | **7.17** | | **5.71** | | **4.01** | | **2.78** | | **2.16** | | **2.24** | | **2.49** | | **3.32** | | **2.36** | | **3.29** | | **3.64** | | **3.98** | | **2.22** | | **3.21** | | **2.33** | | **3.31** | | **3.41** | | **2.20** | | **7.61** | | **2.17** | | 1.39 | | **3.18** | | **3.49** | | **6.29** | | **3.09** | | **2.23** | | **2.44** | | **2.03** | | **2.75** | | **2.87** | | **7.93** | | **2.68** | | **2.28** | | 1.19 | | **2.23** | | **2.42** | | **2.92** | | **2.10** | | **68.36** | | **7.87** | | **6.28** | | **2.93** | | **2.61** | | **2.08** | | **3.51** | | **4.56** | | **2.16** | | **6.97** | | **2.56** | | **3.13** | | **2.53** | | **2.49** | | **2.08** | | **2.87** | | **2.81** | | **2.07** | | **3.01** | | **4.30** | | **2.54** | | **2.50** | | **3.26** | | **3.82** | | **4.12** | | -1.14 | | -1.89 | | **3.05** | | **3.33** | | **2.43** | | **2.43** | | **2.06** | | **3.56** | | **2.28** | | **2.16** | | **2.77** | | **2.09** | | **2.74** | | 1.19 | | -1.02 | | **3.28** | | **3.16** | | **5.65** | | **4.65** | | **2.68** | | **11.27** | | **13.28** | | -1.17 | | **9.75** | | **4.59** | | **4.84** | | **3.14** | | **3.08** | | **2.04** | | **5.69** | | **38.73** | | **3.47** | | **2.35** | | **2.67** | | **3.64** | | **3.93** | | **2.16** | | **2.01** | | **3.67** | | **24.34** | | 1.14 | | **14.59** | | **3.63** | | 1.38 | | **4.15** | | **3.49** | | **2.82** | | **2.42** | | **7.06** | | **5.42** | | **2.17** | | **2.20** | | **2.07** | | **4.34** | | **3.39** | | **2.20** | | **3.36** | | **2.53** | | -1.14 | | **2.82** | | **2.48** | | **2.12** | | **-2.50** | | **2.98** | | **3.05** | | **2.04** | | **2.18** | | **3.15** | | **2.96** | | **3.92** | | **2.22** | | **4.37** | | **2.13** | | **2.24** | | **-3.02** | | **3.55** | | **2.55** | | **2.07** | | **2.84** | | **2.26** | | **5.11** | | **3.00** | | **2.23** | | **4.91** | | **2.60** | | **7.81** | | -1.52 | | -1.39 | | **3.02** | | **3.48** | | **2.32** | | **2.03** | | **2.19** | | **2.15** | | **6.56** | | **2.41** | | **2.43** | | -1.44 | | 1.80 | | -1.30 | | **3.52** | | **2.73** | | **2.15** | | **2.51** | | **2.55** | | **3.25** | | **5.47** | | **2.25** | | **4.99** | | **2.41** | | **2.21** | | **4.13** | | **3.13** | | **3.35** | | 1.56 | | **2.77** | | **2.90** | | **2.07** | | **2.95** | | **2.07** | | **4.08** | | **2.43** | | **3.71** | | **2.67** | | -1.06 | | **2.78** | | -1.12 | | **2.02** | | **3.02** | | **2.44** | | **2.61** | | **4.16** | | **2.97** | | **2.51** | | **3.04** | | **2.23** | | **3.48** | | **2.76** | | **2.27** | | **2.35** | | 1.73 | | **3.12** | | -1.48 | | -1.27 | | **2.08** | | -1.51 | | **6.85** | | **2.22** | | **2.52** | | **-2.83** | | **2.75** | | **2.50** | | 1.65 | | **2.12** | | **-3.11** | | **2.49** | | | 0.02 | | --- | | 0.01 | | 0.03 | | 0.01 | | 0.00 | | 0.02 | | 0.11 | | 0.03 | | 0.01 | | 0.01 | | 0.01 | | 0.00 | | 0.06 | | 0.01 | | 0.01 | | 0.29 | | 0.05 | | 0.00 | | 0.01 | | 0.01 | | 0.02 | | 0.01 | | 0.01 | | 0.02 | | 0.00 | | 0.01 | | 0.01 | | 0.02 | | 0.01 | | 0.00 | | 0.00 | | 0.03 | | 0.01 | | 0.02 | | 0.00 | | 0.00 | | 0.02 | | 0.04 | | 0.20 | | 0.00 | | 0.00 | | 0.01 | | 0.01 | | 0.05 | | 0.01 | | 0.02 | | 0.03 | | 0.10 | | 0.00 | | 0.02 | | 0.02 | | 0.02 | | 0.04 | | 0.03 | | 0.01 | | 0.03 | | 0.02 | | 0.04 | | 0.01 | | 0.02 | | 0.01 | | 0.03 | | 0.02 | | 0.01 | | 0.01 | | 0.04 | | 0.04 | | 0.02 | | 0.01 | | 0.02 | | 0.10 | | 0.01 | | 0.24 | | 0.02 | | 0.00 | | 0.03 | | 0.03 | | 0.03 | | 0.02 | | 0.01 | | 0.04 | | 0.02 | | 0.01 | | 0.04 | | 0.02 | | 0.01 | | 0.01 | | 0.03 | | 0.00 | | 0.00 | | 0.02 | | 0.01 | | 0.03 | | 0.00 | | 0.05 | | 0.03 | | 0.00 | | 0.01 | | 0.02 | | 0.03 | | 0.00 | | 0.02 | | 0.36 | | 0.01 | | 0.04 | | 0.02 | | 0.02 | | 0.02 | | 0.04 | | 0.00 | | 0.02 | | 0.02 | | 0.02 | | 0.02 | | 0.01 | | 0.10 | | 0.04 | | 0.02 | | 0.02 | | 0.02 | | 0.02 | | 0.00 | | 0.01 | | 0.02 | | 0.10 | | 0.05 | | 0.02 | | 0.01 | | 0.01 | | 0.02 | | 0.00 | | 0.01 | | 0.01 | | 0.00 | | 0.03 | | 0.01 | | 0.02 | | 0.01 | | 0.03 | | 0.03 | | 0.04 | | 0.01 | | 0.31 | | 0.02 | | 0.00 | | 0.21 | | 0.03 | | 0.00 | | 0.04 | | 0.02 | | 0.02 | | 0.05 | | 0.01 | | 0.07 | | 0.04 | | 0.03 | | 0.02 | | 0.01 | | 0.00 | | 0.05 | | 0.51 | | 0.00 | | 0.02 | | 0.00 | | 0.01 | | 0.04 | | 0.00 | | 0.02 | | 0.03 | | 0.00 | | 0.05 | | 0.01 | | 0.04 | | 0.02 | | 0.04 | | 0.02 | | 0.02 | | 0.00 | | 0.00 | | 0.01 | | 0.04 | | 0.04 | | 0.01 | | 0.00 | | 0.03 | | 0.02 | | 0.11 | | 0.01 | | 0.21 | | 0.13 | | 0.01 | | 0.00 | | 0.00 | | 0.02 | | 0.02 | | 0.00 | | 0.00 | | 0.00 | | 0.01 | | 0.51 | | 0.06 | | 0.15 | | 0.16 | | 0.03 | | 0.03 | | 0.19 | | 0.13 | | 0.02 | | 0.01 | | 0.01 | | 0.04 | | 0.04 | | 0.00 | | 0.01 | | 0.03 | | 0.02 | | 0.05 | | 0.01 | | 0.01 | | 0.01 | | 0.06 | | 0.04 | | 0.00 | | 0.04 | | 0.04 | | 0.03 | | 0.64 | | 0.00 | | 0.50 | | 0.03 | | 0.02 | | 0.04 | | 0.02 | | 0.03 | | 0.01 | | 0.04 | | 0.01 | | 0.01 | | 0.01 | | 0.00 | | 0.00 | | 0.00 | | 0.11 | | 0.00 | | 0.07 | | 0.13 | | 0.02 | | 0.21 | | 0.01 | | 0.02 | | 0.04 | | 0.04 | | 0.02 | | 0.02 | | 0.22 | | 0.01 | | 0.04 | | 0.02 | |

**Additional file 1:** Table S3. GO categories associated with the inflammatory response in the 0.25 MPa.

| No | GO term | Description | | |
| --- | --- | --- | --- | --- |
| 1  2 | GO:0050727  GO:0050729 | |  | regulation of inflammatory response  positive regulation of inflammatory response |

**Additional file 1:** Table S4. GO categories associated with the inflammatory response in the 0.42 MPa.

| No | GO term | Description | | |
| --- | --- | --- | --- | --- |
| 1  2  3  4  5  6  7  8  9  10  11  12  13  14  15  16  17  18  19  20  21  22 | GO:0006954 GO:0050727 GO:0050729  GO:0002526  GO:0050728  GO:0002675  GO:0002673  GO:0002437  GO:0002532  GO:0150076  GO:0002269  GO:0002523  GO:0002438  GO:0002866  GO:0002863  GO:0002864  GO:0002861  GO:0150077  GO:0002441  GO:0002349  GO:0002246  GO:0090594 | |  | inflammatory response  regulation of inflammatory response  positive regulation of inflammatory response  acute inflammatory response  negative regulation of inflammatory response  positive regulation of acute inflammatory response  regulation of acute inflammatory response  inflammatory response to antigenic stimulus  production of molecular mediator involved in inflammatory response  neuroinflammatory response  leukocyte activation involved in inflammatory response  leukocyte migration involved in inflammatory response  acute inflammatory response to antigenic stimulus  positive regulation of acute inflammatory response to antigenic stimulus  positive regulation of inflammatory response to antigenic stimulus  regulation of acute inflammatory response to antigenic stimulus  regulation of inflammatory response to antigenic stimulus  regulation of neuroinflammatory response  histamine secretion involved in inflammatory response  histamine production involved in inflammatory response  wound healing involved in inflammatory response  inflammatory response to wounding |

**Additional file 1: Table S5. GO categories associated with the NF-**B pathway in the 0.25 MPa.

| No | GO term | Description | | |
| --- | --- | --- | --- | --- |
| 1 | GO:0007253 | |  | cytoplasmic sequestering of NF-kappaB |

**Additional file 1: Table S6. GO categories associated with the NF-**B pathway in the 0.42 MPa.

| No | GO term | Description | | |
| --- | --- | --- | --- | --- |
| 1  2 | GO:0051092  GO:0007249 | |  | positive regulation of NF-kappaB transcription factor activity  I-kappaB kinase/NF-kappaB signaling |

**Additional file 1:** Table S7. Primers used for real-time qRT-PCR

| Gene | Primer | Sequence | | Annealing temperature (℃) | | Product Size (bp) | Number of cycle | Accession Number |
| --- | --- | --- | --- | --- | --- | --- | --- | --- |
| Vimentin | Forward | | CAAGCAGGAGTCAAACGAGT | | 60 | 123 | 45 | NM_011701 |
|  | Reverse | | AGGGCAAAATTCTCTTCCAT | |  |  |  |  |
| GFAP | Forward | | CAGCTGAACTGAACCAGCTT | | 60 | 146 | 45 | NM_001131020 |
|  | Reverse | | CTGTGCAAAGTTGTCCCTCT | |  |  |  |  |
| Lcn2 | Forward | | TGGAAGAACCAAGGAGCTGT | | 60 | 140 | 40 | NM_008491 |
|  | Reverse | | CACACTCACCACCCATTCAG | |  |  |  |  |
| Tgm1 | Forward | | TCAACTCTGCACACGACACA | | 60 | 144 | 45 | NM_019984 |
|  | Reverse | | GCAGATCTGGCCTCTTCATC | |  |  |  |  |
| Serpina3n | Forward | | ACATTGATGGTGCTGGTGAA | | 60 | 152 | 40 | NM_009252 |
|  | Reverse | | TAGGGTGTGGTCAGGTCCTC | |  |  |  |  |
| H2-D1 | Forward | | CATGGTGATCGTTGCTGTTC | | 60 | 126 | 45 | NM_010380 |
|  | Reverse | | CCTGGAGCCAGAGCATAGTC | |  |  |  |  |
| Gbp2 | Forward | | GGAGGAGCTGTGTGGTGAAT | | 60 | 135 | 40 | NM_010260 |
|  | Reverse | | TAGGTCTGCACCAGGCTCTT | |  |  |  |  |
| Emp1 | Forward | | TGCTATCAAGGCAGTGCAAG | | 60 | 124 | 45 | NM_010128 |
|  | Reverse | | GGACCCTGAGAGGAAGAACC | |  |  |  |  |
| Stat3 | Forward | | GAGGAGCTGCAGCAGAAAGT | | 60 | 190 | 40 | NM_011486 |
|  | Reverse | | TCGTGGTAAACTGGACACCA | |  |  |  |  |
| CD68 | Forward | | CCAATTCAGGGTGGAAGAAA | | 60 | 161 | 45 | NM_001291058 |
|  | Reverse | | CTCGGGCTCTGATGTAGGTC | |  |  |  |  |
| Aif1 | Forward | | ATGTCCTTGAAGCGAATGCT | | 60 | 143 | 45 | NM_019467 |
|  | Reverse | | GATCTCTTGCCCAGCATCAT | |  |  |  |  |
| Ccl12 | Forward | | GTCCTCAGGTATTGGCTGGA | | 60 | 181 | 45 | NM_011331 |
|  | Reverse | | GGGTCAGCACAGATCTCCTT | |  |  |  |  |
| Arg1 | Forward | | TCACCTGAGCTTTGATGTCG | | 60 | 134 | 45 | NM_007482 |
|  | Reverse | | CTGAAAGGAGCCCTGTCTTG | |  |  |  |  |
| Bcl2a1b | Forward | | CTCCCTGGCTGAGCACTATC | | 60 | 156 | 40 | NM_007534 |
|  | Reverse | | TCCACGTGAAAGTCATCCAA | |  |  |  |  |
| Icam-1 | Forward | | TGGTGATGCTCAGGTATCCA | | 60 | 147 | 40 | NM_010493 |
|  | Reverse | | GGTCCACTCTCGAGCTCATC | |  |  |  |  |
| Ccr5 | Forward | | ACACCCTGTTTCGCTGTAGG | | 60 | 134 | 40 | NM_009917 |
|  | Reverse | | GAATTCCTGGAAGGTGGTCA | |  |  |  |  |
| Serpine1 | Forward | | GTAGCACAGGCACTGCAAAA | | 60 | 182 | 40 | NM_008871 |
|  | Reverse | | ATCACTTGGCCCATGAAGAG | |  |  |  |  |
| Gpr84 | Forward | | AGGTGACCCGTATGTGCTTC | | 60 | 176 | 40 | NM_030720 |
|  | Reverse | | GTTCATGGCTGCATAGAGCA | |  |  |  |  |
| Psmb8 | Forward | | CAGTCCTGAAGAGGCCTACG | | 60 | 121 | 40 | NM_010724 |
|  | Reverse | | CACTTTCACCCAACCGTCTT | |  |  |  |  |
| Tm4sf1 | Forward | | GCCCAAGCATATTGTGGAGT | | 60 | 112 | 40 | NM_008536 |
|  | Reverse | | GCCTCCAAGCATTCCATTTA | |  |  |  |  |
| CD14 | Forward | | CAAGTGGGGAACCTGTCACT | | 60 | 186 | 40 | NM_009841 |
|  | Reverse | | AAACCAGGAGGATGCAAATG | |  |  |  |  |
| Srgn | Forward | | GCTAATCCAGAGGCTGAGTG | | 60 | 121 | 40 | NM_011157 |
|  | Reverse | | AGCAGGATAACCTTGCACTG | |  |  |  |  |
| Apod | Forward | | CTGGCTCTTCCATGTGGATT | | 60 | 111 | 45 | NM_007470 |
|  | Reverse | | TCGATGTCGATGCCATTAGA | |  |  |  |  |
| C4b | Forward | | GTGGTCGAAGAGCAGGAGTC | | 60 | 145 | 40 | NM_009780 |
|  | Reverse | | AGGTCAGCTTCTCCAGGTCA | |  |  |  |  |
| CD40 | Forward | | CCTGGCTTTGGAGTTATGGA | | 60 | 195 | 40 | NM_011611 |
|  | Reverse | | CCGGGACTTTAAACCACAGA | |  |  |  |  |
| Gbp4 | Forward | | AGCATGAACACCATCAACCA | | 60 | 162 | 40 | NM_008620 |
|  | Reverse | | GAAATCCCGAACAGTCCAGA | |  |  |  |  |

**Additional file 1:** Table S8. The MR parameters used in the study

| **Sequence** | **Purpose** | **TE (ms)** | **TR (ms)** | **FOV**  **(mm^2^)** | **Matrix**  **size** | **Slice Thickness (mm)** | **NEX** |
| --- | --- | --- | --- | --- | --- | --- | --- |
| RARE T2-weighted | Sonication targeting | 33 | 2500 | 25×25 | 256×256 | 1.0 | 3 |
| RARE T1-weighted | Detection of BBB  Disruption | 6.5 | 1500 | 25×25 | 256×256 | 1.0 | 5 |
| RARE  VTR | T1 mapping | 7.5 | 160–  12000 | 25×25 | 256×256 | 1.0 | 3 |
| FLASH  DCE-MRI | Permeability measurement | 1.43 | 24.28 | 35×35 | 128×128 | 1.0 | 3 |
